# Integration of detection and tracking networks for automated rib multiplanar reconstruction: a feasibility study for fracture diagnosis

**DOI:** 10.1186/s41747-026-00703-4

**Published:** 2026-03-17

**Authors:** Donglin Wen, Huasheng Zhuo, Xuedan Zeng, Yi Wang, Guanglan Liao, Tielin Shi, Gang Wu, Zhiyong Liu

**Affiliations:** 1 Department of Radiology, Tongji Hospital of Tongji Medical College of Huazhong University of Science and Technology, Wuhan, China; 2 School of Mechanical Science and Engineering, Huazhong University of Science and Technology, Wuhan, China

**Keywords:** Artificial intelligence, Diagnosis (computer-assisted), Imaging (three-dimensional), Rib fractures, Tomography (x-ray computed)

## Abstract

**Objective:**

Rib fractures are common yet time-consuming to diagnose. This study explores automation *via* multiplanar reconstruction and intelligent detection algorithms to accelerate and optimize clinical assessment.

**Materials and methods:**

A retrospective study was conducted with data from consecutive three-dimensional (3D) computed tomography (CT) examinations of the ribs in 230 patients (137 males), aged 51.7 ± 13.0 years (mean ± standard deviation). Object detection with tracking algorithms and integrated evaluation functions was applied to construct the automatic multiplanar reconstruction (MPR) system. Two readers independently conducted evaluations using automatic multiplanar reconstructions, curved surface reconstructions (CSR), and 3D reconstructions. Results were compared to a reference standard (RS) created by two senior radiologists.

**Results:**

Of 5,520 ribs analyzed, 1,065 (19.3%) were positive at RS. Using automatic MPR, overall 85.4% sensitivity (910/1,065) (95% confidence interval 83.2‒87.5%) and 98.9% specificity (4,406/4,445) (95% CI: 98.5‒99.2%) were obtained. The performance of original CT, CSR, and 3D images was: sensitivity 94.2%, 79.4%, and 58.2%; and specificities 99.6%, 96.2%, and 99.2%, respectively. Reading time decreased by approximately 75% from 159.3 ± 50.5 s using original CT images to 41.2 ± 6.6 s using MPR.

**Conclusion:**

The automatic MPR system offered an accurate solution for diagnosing rib lesions, reducing the reading time. While superior to CSR and 3D reconstructions, automatic MPR should be regarded as a complement to, rather than a substitute for, original CT images in its current form. Future research expanding datasets, exploring different clinical scenarios, and enhancing training for younger physicians is expected.

**Relevance statement:**

Automatic MPR significantly improves rib fracture diagnosis speed and accuracy, reducing evaluation time by 75%. This artificial intelligence system enhances radiologist performance and promises broader clinical integration in trauma care and emergency imaging workflows.

**Key Points:**

- Over 5,500 ribs were analyzed, with 1,065 (19.3%) positive at the reference standard created by two senior radiologists.
- Using automatic MPR, overall 85.4% sensitivity and 98.9% specificity were obtained.
- The reading time decreased by approximately 75% from 159.3 ± 50.5 s using original CT images to 41.2 ± 6.6 s using MPR.

**Graphical Abstract:**

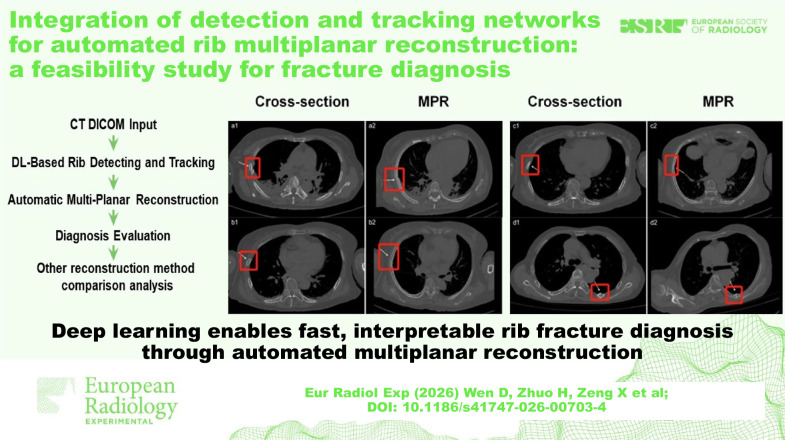

## Background

Chest trauma is a significant global cause of morbidity and mortality, with traffic accidents and falls accounting for a large proportion of cases [1–4]. Such trauma often results in complex injuries involving the lungs, pleura, heart, and ribs [5–7]. Among these, rib fractures are the most common injuries in blunt chest trauma, occurring in 10‒40% of emergency department cases [8]. Accurate and timely diagnosis of rib fractures is essential for both clinical efficacy and medicolegal defensibility [9, 10]. For example, accurate fixation of the chest wall can reduce patient discomfort and prevent long-term complications, including chronic pain or thoracic deformity [11, 12]. It also assists emergency physicians by highlighting regions at risk for serious complications such as pneumothorax and hemothorax in critically ill patients [13, 14]. Furthermore, precise imaging provides objective evidence that helps minimize the likelihood of medical disputes due to underdiagnosis or misinterpretation [15, 16].

Conventional rib fracture diagnosis typically requires clinicians to manually scroll through hundreds of axial CT images, a labor-intensive and time-consuming process [17, 18]. In response, various artificial intelligence and medical image postprocessing techniques have emerged in recent years to accelerate this workflow [19–25]. Some artificial intelligence tools offer automatic detection and localization of rib abnormalities, allowing clinicians to focus on reviewing the results [26, 27]. The postprocessing techniques, like multiplanar reconstruction (MPR), enhance the visualization of the rib cage and the associated lesion, providing more accurate images for rib fracture assessment [28–31]. However, due to the complex and curved anatomy of the rib cage, manual MPR remains laborious and empirically demanding [32], which is undesirable in the acute care setting where rapid diagnosis is critical [33]. Therefore, developing automated MPR systems is essential to improving diagnostic efficiency and accuracy.

In this work, we investigated the diagnostic performance of a novel automatic MPR system utilizing advanced object detection and tracking algorithms to assist in rib lesion diagnosis by comparing it with conventional axial CT interpretation. By accelerating the identification of rib fractures and related injuries, our system aimed to improve the efficiency of trauma assessment and facilitate earlier intervention when clinically warranted.

## Materials and methods

### Dataset

**Fig. 1:**
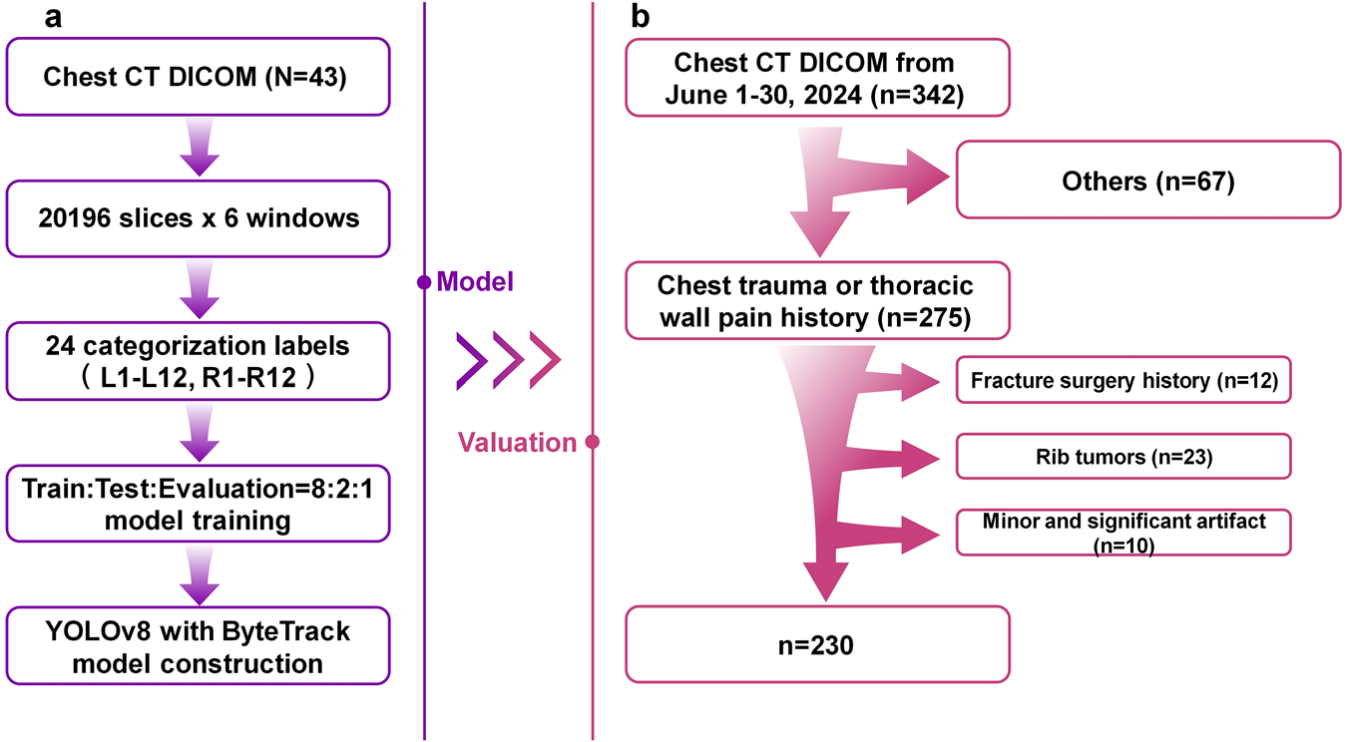
System construction and patient enrollment procedure. **a** System modeling using 10 cases of uninjured ribs. **b** Data collection program for system valuation

The Medical Ethics Committee of Tongji Hospital, Tongji Medical College of HUST, approved this retrospective study. Informed consent was not required for this study. Forty-three healthy cases were used to construct the automatic MPR system, as shown in Fig. 1a. The 43 healthy cases were divided into 20,196 slice images, and with six different window settings applied, the dataset expanded to 121,176 images. Detection box labels were created for 24 ribs. All annotation work was performed by qualified medical professionals with routine experience in musculoskeletal imaging. The annotation process was conducted using LabelImg version 1.8.1 (Tzutalin). The data were split into training, evaluation, and validation sets in an 8:2:1 ratio and used to train models.

The process of enrolling patients is shown in Fig. 1b. A total of 342 consecutive chest CT examinations performed between June 1 and June 30, 2024, were collected. These examinations were requested based on clinical indications suggestive of suspected rib pathology, including rib trauma, rib tumors, thoracic wall pain, and other rib-related conditions. The expected sample was a mixed population requiring diagnostic analysis of rib fractures, in which some patients had rib fractures and others did not. No separate control group was designed, as the study aimed to evaluate diagnostic performance rather than analyze population characteristics. The dataset itself included non-fracture cases, which allowed assessment across both normal and pathological conditions. Patient selection and exclusion criteria were documented in accordance with the CheckList for EvaluAtion of Radiomics research (CLEAR) guideline endorsed by ESR and EuSoMII [34], specifically complying with Item#7 (adherence to guidelines), Item#10 (study nature), Item#11 (eligibility criteria), Item#13 (data source), Item#44 (baseline demographic and clinical characteristics), and Item#45 (flowchart for eligibility criteria).

Inclusion criteria were: consecutive chest CT examinations performed within the study period; clinical history suggestive of suspected rib pathology (*e.g*., chest trauma, thoracic wall pain). Exclusion criteria were: absence of history of chest trauma or thoracic wall pain (*n* = 67, 19.6%); CT scans with significant artifacts affecting diagnostic quality (*n* = 10, 2.9%). The final study cohort included for diagnostic analysis was composed of 230 rib fracture cases (67.2%, mean age: 51.7 ± 13.0 years, 137 males, 93 females). The special cases retained for exploratory analysis were composed of: 12 cases (3.5%) with prior surgical rib fixation; 23 cases (6.7%) with rib tumors; and 10 cases (2.9%) with minor artifacts.

The corresponding MPR and diagnosis evaluation results of special and complex cases are presented in Section 1 of the Supplementary Material.

### CT protocol

Three different CT scanners (SIEMENS SOMATOM Definition CT, Toshiba Aquilion CT System TSX-302A, United Imaging UCT 780) were used, with a tube voltage of 120 kV, automatic tube current adjustment, and a slice thickness of 0.625 to 1.5 mm.

### Technical details of automatic system construction

**Fig. 2:**
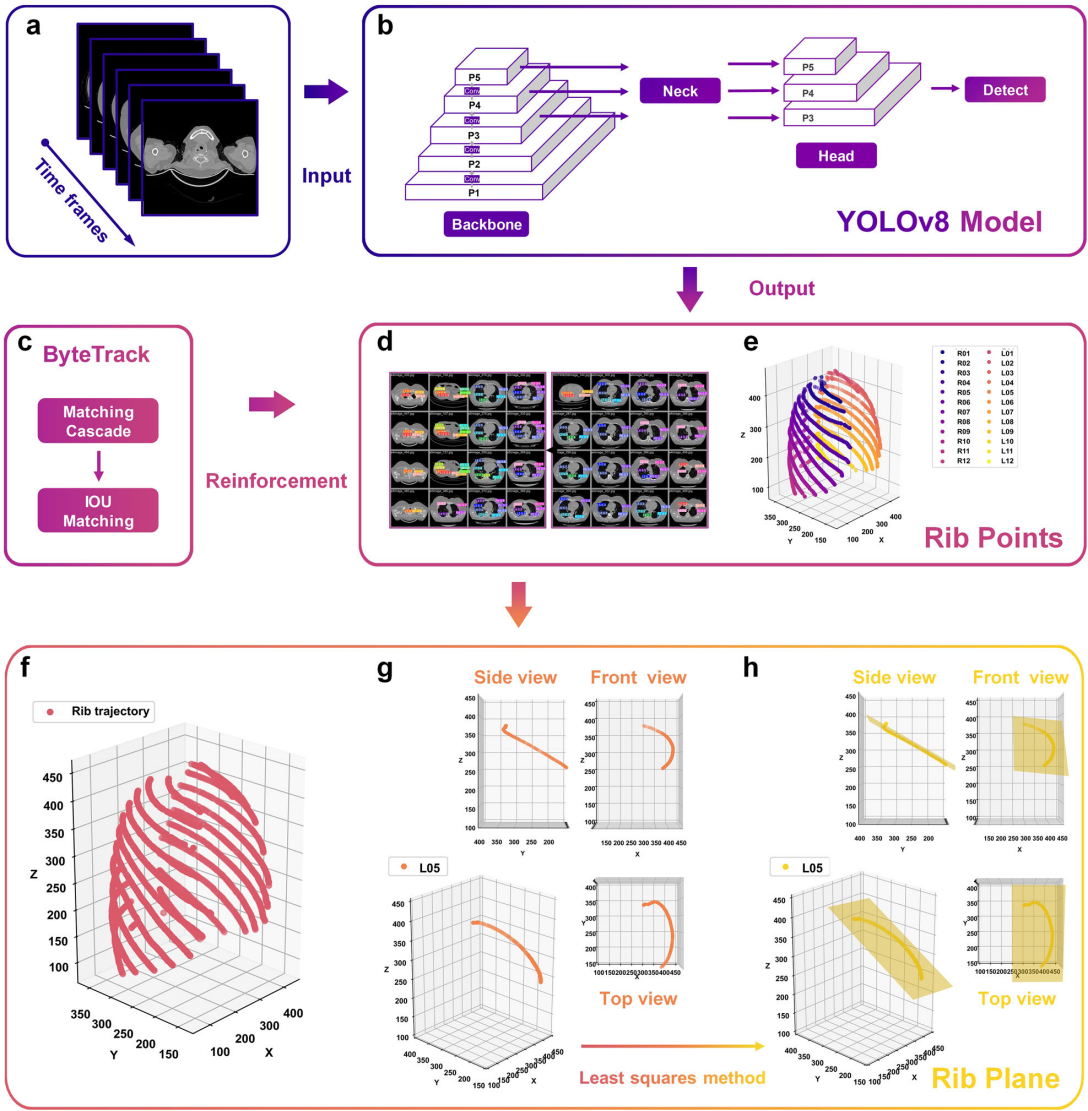
Technical route to accomplish automatic MPR using object detection and tracking algorithms. **a** Converting CT slices into video time frames. **b** YOLOv8 for rib point localization. **c** ByteTrack tracking algorithm runtime logic. **d** Output of YOLOv8 detection of ribs. **e** Ribs localization scattered points. **f** Rib trajectory after tracking algorithm enhancement. **g** Trajectory view of the left fifth rib. **h** Least squares fitting of rib trajectory planes. YOLOv8, You only look once version 8; IOU, Intersection over union

As shown in Fig. 2, this section meticulously outlines the technical workflow of the automatic MPR system. The process began with the conversion of CT slices into sequential video time frames, creating a dynamic representation of the dataset, as shown in Fig. 2a. Utilizing the system model construction method introduced in Fig. 1a, this framework integrated the “You only look once” version 8 object detection algorithm (Ultralytics) (Fig. 2b) and the ByteTrack tracking algorithm (Yifu Zhang) (Fig. 2c), highlighting their distinct roles in model development.

“You only look once” version 8 was employed to precisely identify rib structures within the CT slices, leveraging its advanced object detection capabilities. This enables the extraction of rib center points, which are visually represented in Fig. 2d as highly detailed markers within the slices. These center points, combined with the slice indices, collectively form the three-dimensional (3D) rib scatter point cloud depicted in Fig. 2e. This cloud provides a spatial map where individual ribs are differentiated through a unique visual encoding, revealing their spatial distributions.

ByteTrack further refined the workflow by establishing continuity among rib scatter points. Through its robust tracking mechanism, the algorithm generates seamless trajectories for each rib, as illustrated in Fig. 2f. These trajectories provide the foundation for reconstructing rib planes.

To finalize the process, each rib trajectory underwent computational analysis *via* the least squares method, which optimally fits the scattered data into coherent rib planes. The resulting planes are visualized in Fig. 2g with multiple perspectives (side view, front view, top view) to highlight spatial accuracy and fidelity. The completed MPR process is showcased in Fig. 2h, where rib planes are aligned and fully reconstructed, demonstrating the successful application of this method.

The results of using the object algorithm to detect rib points are provided in Supplementary Material Video 1.

### Model enhancement and fit evaluation functions

To enhance rib detection, a multiwindow data enhancement strategy is proposed that leverages contextual information from various anatomical windows (lung, fat, liver, bone, soft tissue, and vessel). The details of this enhancement technique are provided in Section 2 of the Supplementary Material.

**Fig. 3:**
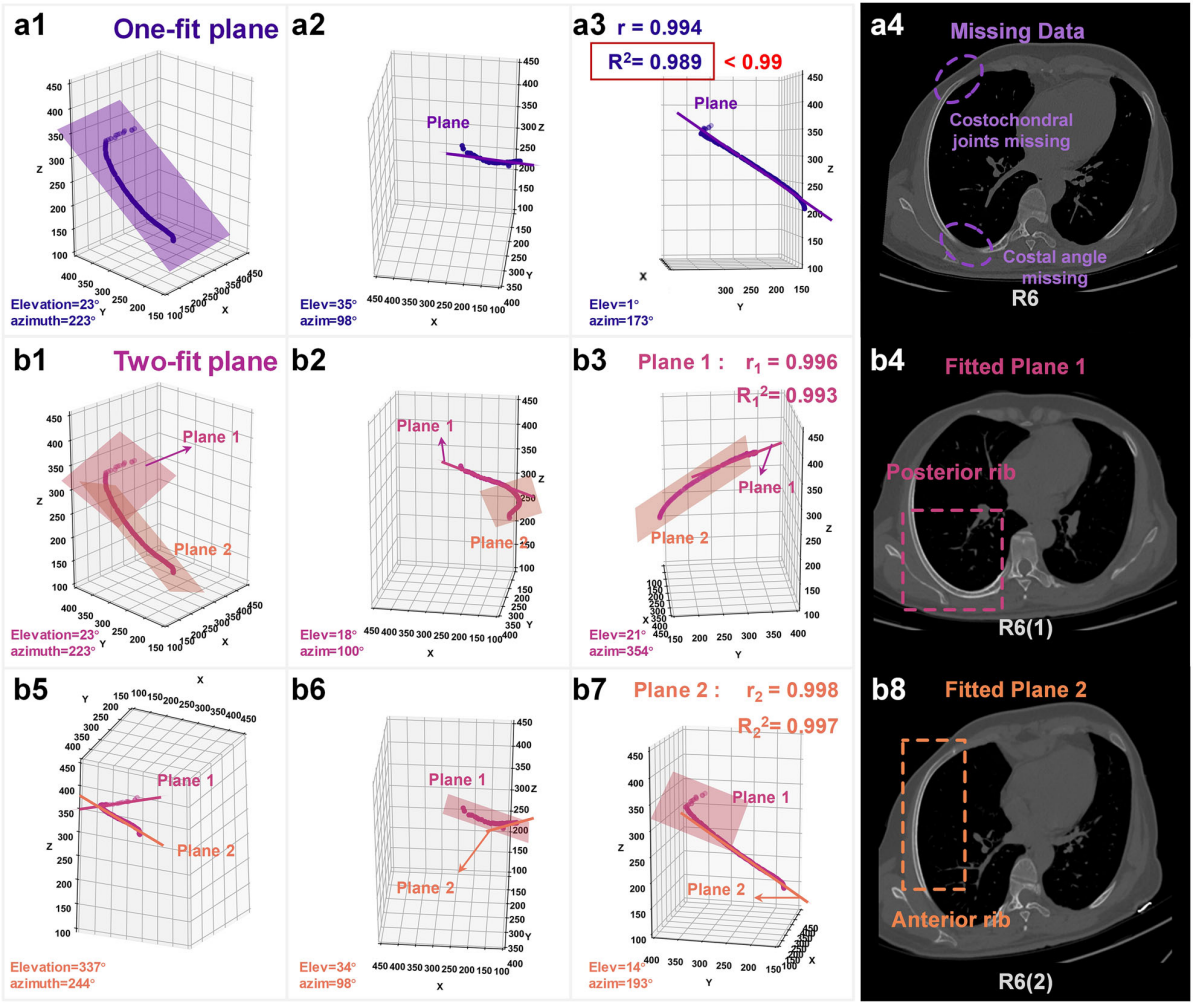
Evaluation of the two-fit plane effect on the right sixth rib. **a1**–**a3** 3D view of one-fit plane at different viewing angles. **a4** MPR image using one-fit plane. **b1**–**b3**, **b5**–**b7** 3D view of two-fit plane at different viewing angles. **b4** MPR image using Plane 1 in the two-fit plane. **b8** MPR image using Plane 2 in the two-fit plane. MPR, Multiplanar reconstruction

Following detection, a multiplane fitting evaluation mechanism is introduced to optimize rib reconstruction. Figure 3a1‒a3 shows the rib plane constructed by the one-fit plane method. Figure 3b1‒b3 shows Plane 1 constructed by the two-fit plane method in a different view, and Fig. 3b5‒b7 shows the results of Plane 2 in the two-fit plane. The MPR images are constructed using a one-fit plane method, which may result in missing data, such as the costochondral joints, costal cartilage, and costal angle (Fig. 3a4). Then the two-fit plane method is needed to complete MPR (Fig. 3b4, b8). This decision needs to be made according to a plane evaluation function. The plane evaluation function is based on the statistical plane linear correlation coefficient *r* and the goodness-of-fit *R*^2^ of the fitted planes. The two-fit plane method is employed to construct the fitting plane when the evaluation metric *r* or *R*^2^ of the single plane is less than a fixed threshold of 0.99.

### Model evaluation and reading methodology

**Fig. 4:**
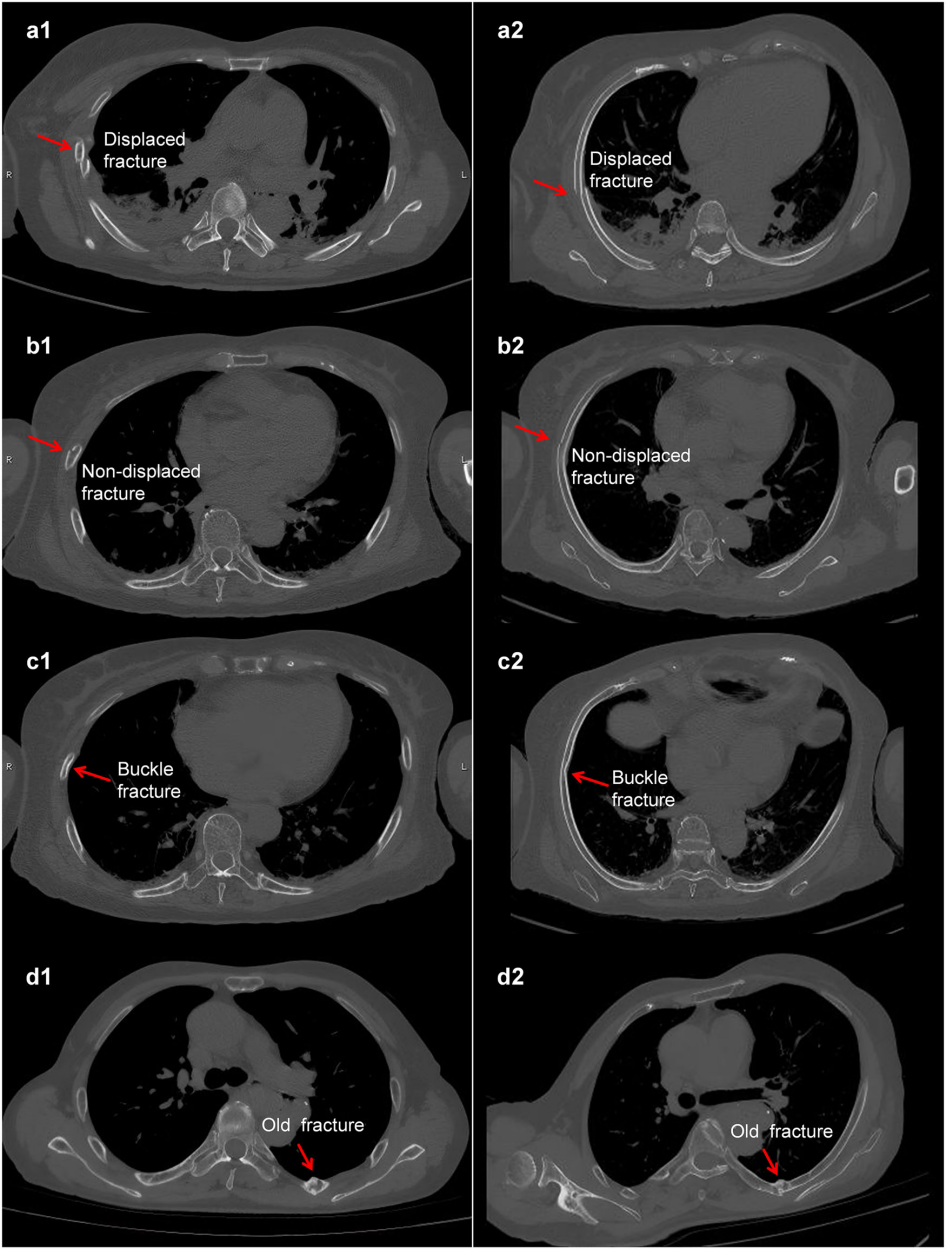
Fracture-type illustrations and corresponding MPR images for each type. **a1**–**d1** Different types of fractures on original CT images. **a2**–**d2** Different types of fractures on the MPR images. The same letter corresponds to the same fracture on the same rib of the same patient. MPR, Multiplanar reconstruction

Rib fractures were adjudicated by two experienced radiologists who were not involved in the creation of training labels. These radiologists reviewed all available modalities, including axial CT, manual and automated MPR, and curved surface reconstruction (CSR), to establish a consensus reference standard for diagnostic evaluation. Subsequently, two other independent junior radiologists, each with approximately 5 years of clinical experience in chest imaging (also not involved in the labeling process) evaluated the automated MPR images in two separate sessions, each spaced at least 1 month apart. The same readers (junior radiologists) also assessed original CT axial slices, CSR reformats, and 3D volume reconstructions for inter-method comparison [23]. Interobserver agreement was quantified using ICC (model 2, single measurement) = 0.857 (95% confidence interval (CI): 0.852–0.866). In case of disagreement, consensus was reached through joint discussion. No formal training session was conducted; however, both readers were familiar with institutional diagnostic criteria and followed routine clinical practice standards for rib fracture evaluation. The example images of rib CSR and 3D reconstruction are provided in Section 3 of the Supplementary Material. The reading process was timed. The diagnostic evaluation was blinded and randomized. In this study, rib fractures were classified into four different pattern categories: displaced, non-displaced, buckle, and old fractures [35, 36]. The fracture-type illustrations and corresponding MPR images for each type are shown in Fig. 4. The system performance evaluation was conducted in three facets: per-rib level, fracture-type level, per-patient level, and demographic information. The sensitivity, specificity, and 95% confidence interval were calculated. Open-source Python (3.8.19), PyTorch (2.5.1), and Deep learning packages were used for detection and statistical analysis. The algorithm and code are available at https://github.com/ultralytics/ultralytics/tree/v8.0.4. C++ (Visual Studio 2022), the VTK library (8.2.0), and the vtk-dicom package were utilized for multiplane reconstruction of DICOM CT files. Complete code and optimal models for experiments and systems are available at https://github.com/Paperdesolate/RibTrack-Yolov8-main.

To enhance reader understanding, a demonstration video illustrating the system’s usage and key functionalities is provided in Supplementary Material Video 2.

### Statistical analysis

To evaluate the diagnostic performance of the proposed image reconstruction strategy, we conducted multi-level statistical analyses based on reference standard annotations and predicted fracture labels. For each rib position, confusion matrix components—true positives, false negatives, true negatives, and false positives—were computed to derive sensitivity and specificity. These metrics were stratified by anatomical side (left *versus* right), rib index (1–12), patient sex, and age group (< 30, 30–50, 50–70, > 70 years). Additionally, lesion-type–specific performance was assessed across five predefined categories (type 0–4), enabling evaluation of the model’s ability to distinguish between varying fracture morphologies.

The stratified analyses were performed at four levels to comprehensively evaluate diagnostic performance. Per-rib analysis treated each of the 24 ribs per patient as an independent unit, enabling assessment of sensitivity and specificity at the finest anatomical granularity. Fracture-type analysis grouped ribs according to reference standard labels (type 0–4), allowing evaluation of the model’s ability to distinguish between different fracture morphologies. Per-patient analysis considered each patient as a single unit, with any rib fracture counted as positive, thereby aligning diagnostic evaluation with clinical decision-making relevance. Demographic analysis stratified results by sex and age categories (< 30, 30–50, 50–70, > 70 years), reflecting potential influences of bone quality and population characteristics on diagnostic accuracy. Age stratification was specifically introduced because osteoporosis and age-related bone fragility substantially increase fracture risk and may alter imaging presentation, thereby affecting diagnostic performance.

For each subgroup, sensitivity and specificity were reported along with 95% confidence intervals estimated using the Wilson score method. Group-wise comparisons of diagnostic sensitivity were performed using Fisher’s exact test for sex-based analysis and the Cochran-Armitage trend test to assess age-related sensitivity variation. Pairwise comparisons between reconstruction methods (MPR *versus* CSR, MPR *versus* 3D) were conducted using McNemar’s test for paired categorical outcomes. For continuous variables such as processing time and confidence scores, repeated-measures ANOVA was applied, followed by Bonferroni correction to adjust for multiple comparisons.

All statistical analyses were performed using Python (pandas, NumPy, SciPy, and statsmodels packages), with a significance threshold set at *p* < 0.05. In addition, a *post hoc* power analysis was conducted on a per-rib basis (*n* = 5,520). Based on the observed distribution of fracture *versus* non-fracture ribs (1,065 *versus* 4,455, 19.3% *versus* 80.7%), the statistical power was estimated to be nearly 100.0% at α = 0.05. This multi-layered evaluation framework was designed to ensure rigorous assessment of diagnostic performance and enhance the interpretability of the reported results across anatomical, demographic, and pathological dimensions. The study design and reporting were guided by principles outlined in CLAIMv2 [37] and METRICS [38].

## Results

### Model performance for rib labeling and automatic MPR

Automatic MPR labeling and reconstruction completeness were evaluated. Labeling referred to rib order. Completeness was assessed by the readers, flagging ribs in the MPR image that were not reconstructed so completely that they could not be used for diagnosis. No errors in order labeling. Complete reconstruction rate at each rib level is shown in Table 1. With the exception of the bilateral first ribs, all ribs showed satisfactory reconstruction, with complete reconstruction rates exceeding 95%. Statistical comparison demonstrated that there was no significant difference between left and right sides at any rib level, and the detailed *p*-values are provided in Table 1. When comparing across rib positions, significant differences were found on both sides (left: χ² = 91.2, df = 11, *p* < 0.001; right: χ² = 63.4, df = 11, *p* < 0.001). The examples of incomplete rib representation images are provided in Section 4 of the Supplementary Material.

**Table 1:** Complete reconstruction rate of automatic multiplanar reconstruction at each rib level, χ^2^ test

	1st	2nd	3rd	4th	5th	6th	7th	8th	9th	10th	11th	12th	*p*-value
Left	90.91%	98.70%	99.13%	97.84%	99.13%	99.13%	97.84%	99.13%	99.57%	99.57%	99.57%	99.57%	< 0.001
Right	92.21%	96.97%	99.57%	96.97%	99.13%	99.13%	97.84%	99.57%	98.70%	99.57%	99.57%	99.57%	< 0.001
*p*-value	0.616	0.201	1.000	0.559	1.000	1.000	1.000	1.000	0.623	1.000	1.000	1.000	‒

### Per-rib level analysis

A total of 5,520 ribs were analyzed in this study; 1,065 ribs (19.3%) were identified as positive by the reference standard, while 959 ribs (17.4%) were diagnosed as positive by the readers using the automatic MPR images. There were 473 rib fractures detected out of 547 reference standards on the left side, with 29 false positives recorded out of 2,213 negative ribs. On the right side, 437 rib fractures were detected out of 518 reference standards, along with 20 false positives recorded from 2,242 negative ribs. Detailed information regarding the total false-positive cases has been added in Section 5 of the Supplementary Materials.

The overall sensitivity and specificity were 85.4% (95% CI: 83.2‒87.5%), 98.9% (95% CI: 98.5‒99.2%). The diagnostic performance of original CT, CSR, and 3D images was evaluated, showing sensitivities of 94.2% (95% CI: 92.6‒95.5%), 79.4% (95% CI: 76.9‒81.8%), and 58.2% (95% CI: 55.2‒61.2%) and specificities of 99.6% (95% CI: 99.4‒99.8%), 96.2% (95% CI: 95.5‒96.7%), and 99.2% (95% CI: 98.9‒99.5%). The details of diagnostic sensitivity and specificity for per-rib level are presented in Table 2 and Section 6 of the Supplementary Material. Across individual ribs, most pairwise comparisons yielded highly significant differences (*p* < 0.001), and all overall comparisons were extremely significant (*p*-values as low as 10⁻⁶⁷); full details are presented in Table S5, Section 8 of the Supplementary Material.

**Table 2:** Sensitivity and specificity evaluated using different methods at the per-rib level, McNemar tests

	MPR image		Original CT image		CSR image		3D image	
	Se (95% CI)	Sp (95% CI)	Se (95% CI)	Sp (95% CI)	Se (95% CI)	Sp (95% CI)	Se (95% CI)	Sp (95% CI)
1st	72.2% (49.1‒87.5%)	99.7% (98.7‒100%)	88.8% (67.2‒96.9%)	99.7% (98.7‒100%)	72.2% (49.1‒87.5%)	99.5% (98.4‒99.9%)	11.1% (3.1‒32.8%)	100% (99.1‒100.0%)
2nd	58.4% (45.1‒70.7%)	99.5% (98.2‒99.9%)	86.7% (75.2‒93.5%)	99.7% (98.6‒100%)	81.1% (68.6‒89.4%)	95.5% (93.1‒97.2%)	50.9% (37.9‒63.9%)	99.7% (98.6‒100.0%)
3rd	85.4% (78.0‒90.7%)	97.9% (95.8‒99.0%)	94.8% (89.3‒97.6%)	99.7% (98.4‒99.9%)	76.0% (67.6‒82.9%)	92.1% (88.8‒94.5%)	60.6% (51.6‒69.1%)	99.1% (97.5‒99.7%)
4th	85.0% (78.2‒90.0%)	97.8% (95.6‒98.9%)	95.7% (91.0‒98.0%)	98.7% (96.8‒99.5%)	79.2% (71.8‒85.2%)	91.5% (88.0‒94.1%)	60.0% (51.7‒67.7%)	98.1% (96.0‒99.1%)
5th	88.4% (82.7‒92.5%)	97.6% (95.2‒98.8%)	95.7% (91.5‒97.9%)	98.6% (96.6‒99.5%)	78.7% (71.9‒84.3%)	95.9% (93.0‒97.7%)	64.2% (56.7‒71.2%)	99.6% (98.1‒99.9%)
6th	90.1% (84.1‒94.0%)	99.3% (97.7‒99.8%)	96.4% (92.0‒98.5%)	100.0% (98.8‒100.0%)	84.5% (77.7‒89.5%)	93.7% (90.5‒95.9%)	60.5% (52.3‒68.2%)	97.1% (94.7‒98.5%)
7th	85.0% (78.1‒90.1%)	99.0% (97.3‒99.7%)	93.2% (87.7‒96.4%)	100.0% (98.8‒100.0%)	75.3% (67.4‒81.9%)	93.5% (90.4‒95.7%)	53.7% (45.3‒62.0%)	99.3% (97.8‒99.8%)
8th	86.4% (78.5‒91.7%)	98.0% (96.0‒99.0%)	95.1% (89.1‒97.9%)	99.4% (98.0‒99.8%)	80.5% (71.9‒87.1%)	96.6% (94.2‒98.1%)	59.2% (49.6‒68.2%)	98.5% (96.8‒99.4%)
9th	88.7% (80.5‒93.8%)	98.3% (96.5‒99.3%)	92.1% (84.6‒96.1%)	99.7% (98.5‒100.0%)	78.6% (69.0‒85.9%)	95.9% (93.4‒97.5%)	52.8% (42.5‒62.8%)	98.3% (96.5‒99.3%)
10th	84.6% (72.5‒92.0%)	99.0% (97.5‒99.6%)	90.3% (79.4‒95.8%)	99.5% (98.2‒99.9%)	82.6% (70.3‒90.6%)	96.5% (94.3‒97.9%)	53.8% (40.5‒66.7%)	100.0% (99.1‒100.0%)
11th	90.3% (75.1‒96.7%)	99.7% (98.7‒100.0%)	93.5% (79.3‒98.2%)	100.0% (99.1‒100.0%)	80.6% (63.7‒90.8%)	99.5% (98.3‒99.9%)	67.7% (50.1‒81.4%)	99.7% (98.7‒100.0%)
12th	90.4% (71.1‒97.3%)	99.5% (98.4‒99.9%)	95.2% (77.3‒99.2%)	99.7% (98.7‒100.0%)	85.7% (65.4‒95.0%)	99.7% (98.7‒100.0%)	71.4% (50.0‒86.2%)	100.0% (99.1‒100.0%)

*CSR* Curved surface reconstruction, *MPR* Multiplanar reconstruction, *Se* Sensitivity, *Sp* Specificity

### Fracture-type level analysis

Of the 1,065 fractures defined as positive by the reference standard, 207 were displaced, 149 non-displaced, 228 buckle, and 481 old fractures. Among the 959 fractures identified as positive by the readers using automated MPR images, 197 were displaced, 114 non-displaced, 202 buckle, and 446 old fractures. The sensitivities of the four types above were 92.7% (95% CI: 88.4‒95.6%), 67.1% (95% CI: 59.2‒74.1%), 66.6% (95% CI: 60.3‒72.5%) and 89.8% (95% CI: 86.8‒92.2%), while the specificities were 99.9% (95% CI: 99.8‒100%), 99.7% (95% CI: 99.6‒99.8%), 99.0% (95% CI: 98.8‒99.3%) and 99.7% (95% CI: 99.5‒99.8%). The details of the classification diagnosis comparison between MPR and the other three image types are shown in Table 3. Pairwise McNemar tests revealed statistically significant differences among reconstruction methods across all fracture types, with *p*-values often below 10⁻¹⁵. Notably, no significant difference was observed between MPR and Original CT for displaced fractures (*p* = 0.146) and between CSR and 3D for non-displaced fractures (*p* = 0.864). Nevertheless, original CT images remained the most accurate modality overall, particularly for challenging non-displaced fractures, underscoring the complementary role of MPR reconstructions. Full results are provided in Table S6, Section 8 of the Supplementary Material.

**Table 3:** Sensitivity and specificity evaluated using different methods at the fracture-type level

		MPR image	Original CT image	CSR image	3D image
Displaced fractures	Se (95% CI)	92.7% (88.4‒95.6%)	98.5% (95.8‒99.5%)	68.1% (61.5‒74.1%)	82.6% (76.9‒87.2%)
	Sp (95% CI)	99.9% (99.8‒100.0%)	99.8% (99.7‒99.9%)	99.7% (99.6‒99.9%)	98.5% (98.2‒98.9%)
Non-displaced fractures	Se (95% CI)	67.1% (59.2‒74.1%)	79.1% (72.0‒84.9%)	45.6% (37.8‒53.6%)	0.67% (0.1‒3.7%)
	Sp (95% CI)	99.7% (99.6‒99.8%)	99.7% (99.5‒99.8%)	98.6% (98.3‒99.0%)	99.9% (99.9‒100.0%)
Buckle fractures	Se (95% CI)	66.6% (60.3‒72.5%)	81.1% (75.6‒85.7%)	30.7% (25.1‒37.0%)	0.0% (0.0‒1.7%)
	Sp (95% CI)	99.0% (98.8‒99.3%)	99.4% (99.2‒99.6%)	97.1% (96.7‒97.6%)	100.0% (99.9‒100.0%)
Old fractures	Se (95% CI)	89.8% (86.8‒92.2%)	93.7% (91.2‒95.6%)	87.3% (84.0‒90.0%)	64.8% (60.5‒69.0%)
	Sp (95% CI)	99.7% (99.5‒99.8%)	99.8% (99.7‒99.9%)	98.3% (98.0‒98.7%)	98.1% (97.7‒98.5%)

*CSR* Curved surface reconstruction, *MPR* Multiplanar reconstruction

### Demographic information analysis

The patients were divided into four age groups: under 30 years (12 patients), 30‒49 years (70 patients), 50‒69 years (128 patients), and 70 years or older (20 patients). The sensitivities for these groups were 77.4% (95% CI: 60.2‒88.6%), 82.4% (95% CI: 77.1‒86.6%), 86.9% (95% CI: 84.2‒89.2%) and 85.2% (95% CI: 76.3‒91.2%), and the specificities were 100.0% (95% CI: 98.8‒100.0%), 99.2% (95% CI: 98.5‒99.5%), 98.6% (95% CI: 98.1‒99.0%) and 98.9% (95% CI: 96.8‒99.6%), respectively. The patients were divided by gender (male-to-female ratio: 137:93), with sensitivities of 86.3% (95% CI: 82.5‒89.4%) and 85.0% (95% CI: 82.1‒87.4%), and specificities of 99.1% (95% CI: 98.5‒99.4%) and 98.8% (95% CI: 98.3‒99.1%) for males and females. Table 4 presents the demographic analysis comparing MPR with the other three image types. No statistically significant difference in diagnostic sensitivity was observed between male and female patients across all reconstruction methods (Fisher’s exact test, all *p* ≥ 0.220). For MPR images, diagnostic sensitivity increased from younger to middle-aged patients, reflecting a shift from subtler fractures in the young to more linear, readily detectable fractures in middle age, but then declined slightly in those over 70. This non-monotonic trend was not statistically significant (Cochran-Armitage trend test, *p* = 0.086). Detailed *p*-values for sex- and age-based comparisons are provided in Supplementary Table S7.

**Table 4:** Sensitivity and specificity evaluated using different methods for demographic information

				Original CT image		CSR image		3D image	
		Se (95% CI)	Sp (95% CI)	Se (95% CI)	Sp (95% CI)	Se (95% CI)	Sp (95% CI)	Se (95% CI)	Sp (95% CI)
Age	≤ 30	77.4% (60.2‒88.6%)	100.0% (98.8‒100.0%)	90.3% (75.1‒96.7%)	100.0% (98.8‒100%)	58.1% (40.8‒73.6%)	98.4% (96.2‒99.3%)	29.0% (16.1‒46.6%)	100.0% (98.8‒100.0%)
	31‒50	82.4% (77.1‒86.6%)	99.2% (98.5‒99.5%)	92.2% (88.2‒95.0%)	99.7% (99.2‒99.9%)	74.2% (68.3‒79.3%)	98.0% (97.2‒98.6%)	53.3% (47.0‒59.4%)	99.5% (99.0‒99.8%)
	51‒70	86.9% (84.2‒89.2%)	98.6% (98.1‒99.0%)	95.0% (93.1‒96.4%)	99.6% (99.2‒99.8%)	81.5% (78.4‒84.2%)	94.7% (93.7‒95.5%)	60.1% (56.4‒63.7%)	99.0% (98.5‒99.3%)
	> 70	85.2% (76.3‒91.2%)	98.9% (96.8‒99.6%)	94.3% (87.4‒97.5%)	99.3% (97.4‒99.8%)	85.2% (76.3‒91.2%)	96.3% (93.4‒98.0%)	67.0% (56.7‒76.0%)	98.9% (96.8‒99.6%)
Sex	Female	86.3% (82.5‒89.4%)	99.1% (98.5‒99.4%)	95.3% (92.8‒97.0%)	99.7% (99.4‒99.9%)	78.3% (73.9‒82.1%)	96.3% (95.3‒97.0%)	60.7% (55.8‒65.5%)	99.2% (98.7‒99.5%)
	Male	85.0% (82.1‒87.4%)	98.8% (98.3‒99.1%)	93.5% (91.4‒95.1%)	99.5% (99.2‒99.7%)	80.1% (76.9‒82.9%)	96.1% (95.3‒96.8%)	56.8% (53.0‒60.5%)	99.2% (98.8‒99.5%)

*CSR* Curved surface reconstruction, *MPR* Multiplanar reconstruction, *Se* Sensitivity, *Sp* Specificity

### Per-patient level analysis

Compared to the reference standard, 114/230 patients (49.6%) had an exact number match in rib fracture diagnosis using MPR images, while 81/230 patients (35.2%) had either one missed (*n* = 60) or one false-positive rib fracture (*n* = 21). In addition, 23/230 patients (10.0%) had two missed fractures, and 4/230 patients (1.7%) had two false-positive fractures. The error distribution in the number of rib fractures by different methods at the per-patient level is provided in Section 7 of the Supplementary Material.

### Reconstruction and reading time

As detailed in Table 5, the average reading times were 41.2 ± 6.6 s for MPR images, 159.3 ± 50.5 s for original CT images, 29.6 ± 5.1 s for CSR images, and 35.2 ± 7.5 s for 3D images. This difference translates to a reduction of approximately 118 s, or nearly a 75% decrease in reading time.

**Table 5:** Reading time evaluated using different methods

	MPR image	Original CT image	CSR image	3D image
Reader 1	42.9 ± 6.8 s	154.3 ± 50.6 s	28.8 ± 4.5 s	36.2 ± 6.3 s
Reader 2	39.6 ± 6.0 s	164.4 ± 51.2 s	30.3 ± 5.6 s	34.3 ± 8.6 s
Average values	41.2 ± 6.6 s	159.3 ± 50.5 s	29.6 ± 5.1 s	35.2 ± 7.5 s

*CSR* Curved surface reconstruction, *MPR* Multiplanar reconstruction

## Discussion

In this study, we developed and tested an automatic MPR system for ancillary diagnosis of rib lesions using advanced detection and tracking algorithms. The system was validated on 230 chest CT examinations, achieving 100% labeling accuracy, over 95% reconstruction completeness for most ribs, and per-rib sensitivity and specificity of 85.4% and 98.9%, respectively, while reducing reading time by approximately 75%. It generated high-quality MPR images in s with an *R*² goodness-of-fit above 0.99. Beyond simply automating rib-aligned MPR generation, the proposed system integrates detection, labeling, and standardized reconstruction in a unified workflow designed to enhance diagnostic consistency and efficiency across clinical settings.

Unlike previous reports, our method of tracking ribs and automating MPR *via* video stream detection is both novel and comprehensive [18, 23, 39]. The output images from our system were evaluated at multiple levels and compared against other reconstruction techniques. Overall, our MPR system achieved excellent reconstruction completeness across nearly all ribs and demonstrated robust per-rib diagnostic performance, effectively supporting radiologists in reducing false positives during image interpretation. The lower completeness for the first rib is due to its near-horizontal orientation and minimal tilt, which make single-plane fitting under planar evaluation criteria challenging [40]. In addition, the first rib is relatively short, wide, highly curved, and flat in shape, leading to partial edge omissions and greater diagnostic difficulty [41, 42]. In the oldest group, reduced bone density and osteoporosis diminish contrast between lesions and surrounding tissue, leading to lower detection sensitivity. Compared with alternative reconstruction approaches, original CT scans remain the most diagnostically reliable, owing to their comprehensive cross-sectional detail and radiologists’ familiarity with axial views. Compared with CSR and 3D techniques, MPR images demonstrated significantly superior diagnostic consistency (McNemar test, *p* < 0.001 for both comparisons), while preserving anatomical fidelity without introducing the distortions typical of stretched planar reformats or the detail loss inherent to volumetric renderings. This balance of clarity and interpretability made MPR especially effective for accurate fracture detection and classification across different lesion types. Ultimately, our automated MPR system delivers superior performance and efficiency, making it a valuable tool for rib fracture assessment.

Time-motion studies have reported that manual review of rib fractures takes approximately 15–20 min per patient in busy trauma centers, resulting in reporting delays of up to 2–3 h for critical chest trauma findings [43, 44]. Such delays have been associated with a 20% increase in time to chest tube placement and longer stays in the intensive care unit, underscoring the clinical urgency of workflow acceleration [45]. In modern PACS viewers, experienced radiologists can generate a simple rib MPR for a single rib in a few seconds. Completing all 24 ribs manually typically takes anywhere from tens of seconds to several minutes, depending on the user’s skill. In our evaluation, it took 2‒5 min for physicians to evaluate each case using conventional axial CT slices and 5‒15 min to generate high-quality MPRs for all 24 ribs without prior training. Moreover, the PACS workflow does not preserve the manipulated viewing positions: once a rib is reconstructed and the user moves on, those settings are lost, and revisiting any previous rib forces a complete redo of the reconstruction. As a result, consistently producing high-quality manual reconstructions requires progressively more time and effort. Automating this manual process not only eliminates repetitive work burden but also frees clinicians to focus on interpretation, thereby improving overall efficiency.

The limitations of this study include its single-center sample, necessitating the inclusion of multicenter data to mitigate bias. Additionally, due to the requirement for manual radiologist interpretation, the sample size remains insufficient, warranting future expansion. Additionally, while the current system enables precise rib localization through oblique MPR reconstruction, it does not yet support slice thickness adjustment within the reconstruction plane or maximum intensity projection. This limitation stems from the technical difficulty of quantifying slice thickness along curved anatomical trajectories, which may increase interpretation complexity. Another common limitation in chest CT is respiratory artifact, which can obscure fracture visualization. Although our current system does not directly address this issue, future integration of time-motion techniques may help mitigate such artifacts and will be considered in subsequent iterations. These considerations underscore the need for further integration with PACS environments and optimization of routine diagnostic workflows. In future iterations, we also aim to incorporate enhanced interactive features such as *z*-axis scrolling and rib-specific image export to improve clinical usability and compatibility with standard viewers.

These findings filled a gap in existing research, offering an efficient and accurate MPR solution. This work sets the stage for the broader use of automatic imaging technology, enhancing emergency trauma care and radiology.

## Supplementary information

**Additional file 1**: **Section 1:** MPR and diagnosis evaluation results of special and complex cases. **Table S1:** Sensitivities and specificities of the special and complex categories. **Fig. S1:** Representative MPR images of different categories of rib lesions. **(a)** Axial CT image affected by significant artifacts, **(b)** corresponding MPR reconstruction. **(c)** Axial CT image showing a rib tumor, **(d)** corresponding MPR reconstruction. **(e)** Axial CT image of a postoperative rib, **(f)** corresponding MPR reconstruction. **Section 2:** Model enhancement using multiple windows. **Table S2:** Detailed data of model performance by multiwindow enhancement methods. **Fig. S2:** Model performance improvement by multiwindow enhancement methods. **(a)** Model metrics and their dataset performance under six window widths (WW) and window levels (WL), lung: 1500WW, -500WL; fat: 200WW, -100WL; liver: 120WW, 60WL; bone: 2000WW, 300WL; soft tissue: 400WW, 40WL; and vessel: 700WW, 300WL. **(b)** Expected model performance. **(c)** Multi-window enhanced model performance. **Section 3** CSR and 3D images of ribs. **Fig. S3:** CSR image of a patient with blue arrows showing reconstructed edge jaggedness. **Fig. S4:** 3D reconstructed images of the ribs of the same patient. **Section 4** MPR images with incomplete representation of ribs. **Fig. S5: (a)** and **(b)** are single-figure reconstructions of the complete right 1st and 4th ribs, and **(c1)** and **(c2)** are two-figure reconstructions of the complete left 6th rib. As presented in the figure is considered by the reader to be reconstructed completely. **Fig. S6:** Incomplete reconstruction of ribs (white arrows). **(a)** The loss of the middle of the first rib on the right side. **(b)** The partial absence of the anterior rib. **(c)** The partial absence of the posterior rib. All of the above is defined as incomplete reconstruction. **Section 5** MPR and cross-sectional images of false-positive cases. **Fig. S7:** MPR and cross-sectional images of 10 false-positive cases: Patient No.0-9. **Fig. S8:** MPR and cross-sectional images of 10 false-positive cases: Patient No.10-19. **Fig. S9:** MPR and cross-sectional images of 10 false-positive cases: Patient No.20-26. **Fig. S10:** MPR and cross-sectional images of 10 false-positive cases: Patient No.27-34. **Fig. S11:** MPR and cross-sectional images of 10 false-positive cases: Patient No.35-40. **Section 6** Sensitivity and specificity using different methods at per-rib level. **Table S3:** Sensitivity and specificity evaluated using different methods at the left, right and per-level of ribs level. **Section 7** Error distribution in the number of rib fractures by different methods. **Table S4:** Error distribution in the number of rib fractures by different methods at the per-patient level. **Section 8** Statistical analysis of significance testing. **Table S5**: Pairwise McNemar p-values across rib levels and reconstruction methods. **Table S6**: Pairwise McNemar p-values across fracture types and reconstruction methods. **Table S7:** Statistical comparison of diagnostic sensitivity across sex and age groups for each reconstruction method.

Video 1

Video 2

## Data Availability

The data generated during the current study are available from the corresponding author on reasonable request.

## Abbreviations

3D: Three-dimensional
CI: Confidence interval
CSR: Curved surface reconstruction
CT: Computed tomography
MPR: Multiplanar reconstruction

## Code availability

The code generated during the current study is available from the corresponding author on reasonable request.
