# Supplementary material for: Integration of detection and tracking networks for automated rib multiplanar reconstruction: a feasibility study for fracture diagnosis
Source: Eur Radiol Exp. 2026 Mar 17;10:34. doi: 10.1186/s41747-026-00703-4 (PMC12996475; doi:10.1186/s41747-026-00703-4)
Supplement: Supplementary file 1 — Additional file 1: Section 1: MPR and diagnosis evaluation results of special and complex cases. Table S1: Sensitivities and specificities of the special and complex categories. Fig. S1: Representative MPR images of different categories of rib lesions. (a) Axial CT image affected by significant artifacts, (b) corresponding MPR reconstruction. (c) Axial CT image showing a rib tumor, (d) corresponding MPR reconstruction. (e) Axial CT image of a postoperative rib, (f) corresponding MPR reconstruction. Section 2: Model enhancement using multiple windows. Table S2: Detailed data of model performance by multiwindow enhancement methods. Fig. S2: Model performance improvement by multiwindow enhancement methods. (a) Model metrics and their dataset performance under six window widths (WW) and window levels (WL), lung: 1500WW, -500WL; fat: 200WW, -100WL; liver: 120WW, 60WL; bone: 2000WW, 300WL; soft tissue: 400WW, 40WL; and vessel: 700WW, 300WL. (b) Expected model performance. (c) Multi-window enhanced model performance. Section 3 CSR and 3D images of ribs. Fig. S3: CSR image of a patient with blue arrows showing reconstructed edge jaggedness. Fig. S4: 3D reconstructed images of the ribs of the same patient. Section 4 MPR images with incomplete representation of ribs. Fig. S5: (a) and (b) are single-figure reconstructions of the complete right 1st and 4th ribs, and (c1) and (c2) are two-figure reconstructions of the complete left 6th rib. As presented in the figure is considered by the reader to be reconstructed completely. Fig. S6: Incomplete reconstruction of ribs (white arrows). (a) The loss of the middle of the first rib on the right side. (b) The partial absence of the anterior rib. (c) The partial absence of the posterior rib. All of the above is defined as incomplete reconstruction. Section 5 MPR and cross-sectional images of false-positive cases. Fig. S7: MPR and cross-sectional images of 10 false-positive cases: Patient No.0-9. Fig. S8: MPR and cross-sectional images [file 41747_2026_703_MOESM1_ESM.pdf]

Integration of detection and tracking networks for automated rib multiplanar reconstruction: a feasibility study for fracture diagnosis

ELECTRONIC SUPPLEMENTARY MATERIAL

Table of contents:

Section	Title
1	MPR and diagnosis evaluation results of special and complex cases
2	Model enhancement using multiple windows
3	CSR and 3D images of ribs
4	MPR images with incomplete representation of ribs
5	MPR and cross-sectional images of false-positive cases
6	Sensitivity and specificity using different methods at per-rib level
7	Error distribution in the number of rib fractures by different methods
8	Statistical analysis of significance testing

Section 1: MPR and diagnosis evaluation results of special and complex cases

For the remaining 45 cases in other categories, excluding 2 images with significant artifacts, the diagnoses were classified, and the diagnostic performance using MPR images was calculated. Unlike for fractures, we only categorized ribs as negative or positive, using the assessment results of senior radiologists as the reference standard to calculate sensitivity and specificity. For postoperative patients, the operated ribs are defined as positive, while the remaining unoperated ribs are defined as negative. Sensitivities and specificities for minor artifacts (n=8), tumor patients (n=23), and postoperative patients (n=12) were presented in Table S1. Fig. S1 illustrates MPR images of different categories of rib lesions.

Table S1: Sensitivities and specificities of the special and complex categories.

	Minor artifacts	Tumor patients	Postoperative patients	Overall
Sensitivity	80.0% (95CI: 62.7%, 90.5%)	85.2% (95CI: 77.0%, 91.2%)	100% (95CI: 89.1%, 100%)	87.8% (95% CI: 79.2%, 93.5%)
Specificity	83.3% (95CI: 66.4%, 93.4%)	97.1% (95CI: 94.7%, 98.6%)	100% (95CI: 98.6%, 100%)	94.9% (95% CI: 90.9%, 97.4%)

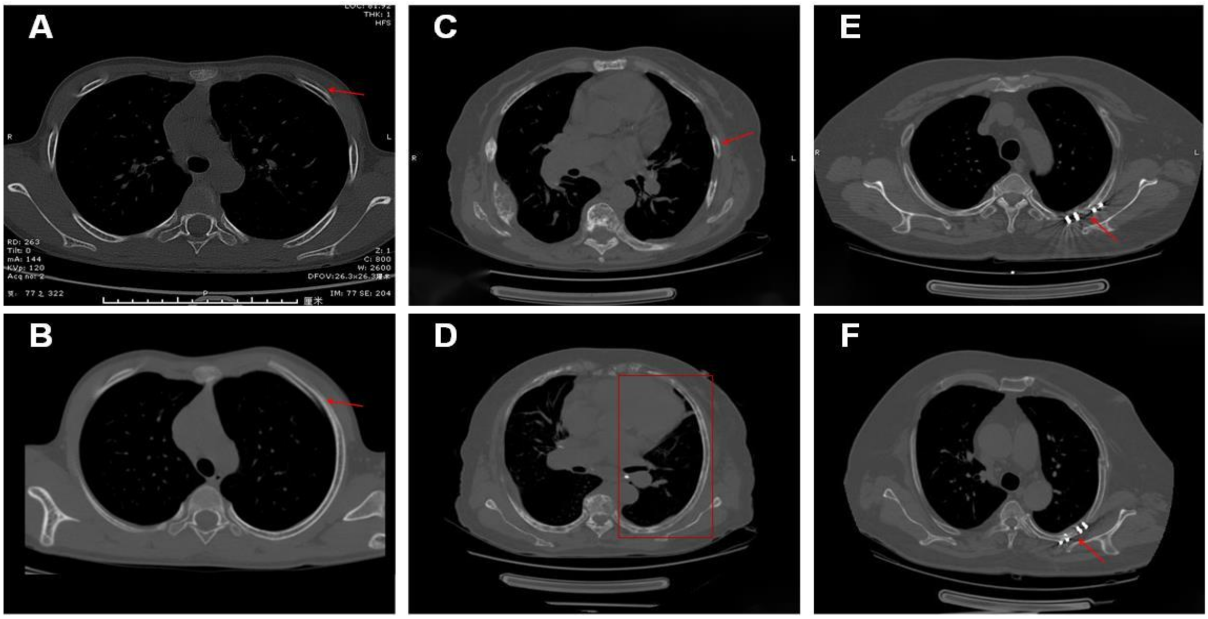

Fig. S1: Representative MPR images of different categories of rib lesions. (a) Axial CT image affected by significant artifacts, **(b)** corresponding MPR reconstruction. **(c)** Axial CT image showing a rib tumor, **(d)** corresponding MPR reconstruction. **(e)** Axial CT image of a postoperative rib, **(f)** corresponding MPR reconstruction.

Section 2: Model enhancement using multiple windows

The original CT images are first processed to generate sub-images with different windowing parameters, highlighting specific tissue characteristics. Independent detection models are constructed for each window using a convolutional neural network, and their performance is evaluated through key metrics. The complementary information from all windows is then fused into an integrated multi-window model, which significantly improves detection accuracy and robustness while maintaining high inference speed. This optimized model is incorporated into our automatic MPR system to provide precise rib localization under challenging imaging conditions.

The tissues and organs surrounding the ribs can provide references for detecting the ribs. As shown in Fig. S2a and Table S2, the models are constructed under the lung, fat, liver, bone, soft tissue, and blood vessel windows. Six metrics about accuracy and inference speed are compared. The model performance under the vessel window is closest to the desired model (Fig. S2b), achieving a Precision of 86.1%, Recall of 88.2%, mAP50 of 91.0%, mAP50-95 of 50.4%, and F1-score of 87.1%. While ensuring an inference speed of 1.2 ms, the multi-window model (Fig. S2c), which integrates data from all six windows, significantly improves accuracy. The model is incorporated into our system as the optimal model, which shows an accuracy of 95.9%, a recall of 93.4%, a mAP50 of 96.8%, and an F1 score of 94.6%.

Table S2: Detailed data of model performance by multi-window enhancement methods.

Model	Precision	Recall	mAP50	mAP50-95	F1-score	Infer_time
Lung	56.1%	77.6%	64.7%	32.8%	65.1%	3.2 ms
Fat	69.2%	65.9%	68.3%	32.4%	67.5%	1.6 ms
Liver	88.3%	82.8%	88.7%	50.1%	85.5%	3.0 ms
Bone	76.3%	82.9%	83.1%	46.7%	79.5%	1.6 ms
Soft-tissue	79.6%	85.4%	86.6%	48.2%	82.4%	2.0 ms
Vessel	86.1%	88.2%	91.0%	50.4%	87.1%	1.2 ms
Muti-window	95.9%	93.4%	96.8%	58.0%	94.6%	1.2 ms

Expectation	100.0%	100.0%	100.0%	60%	100.0%	1.0 ms

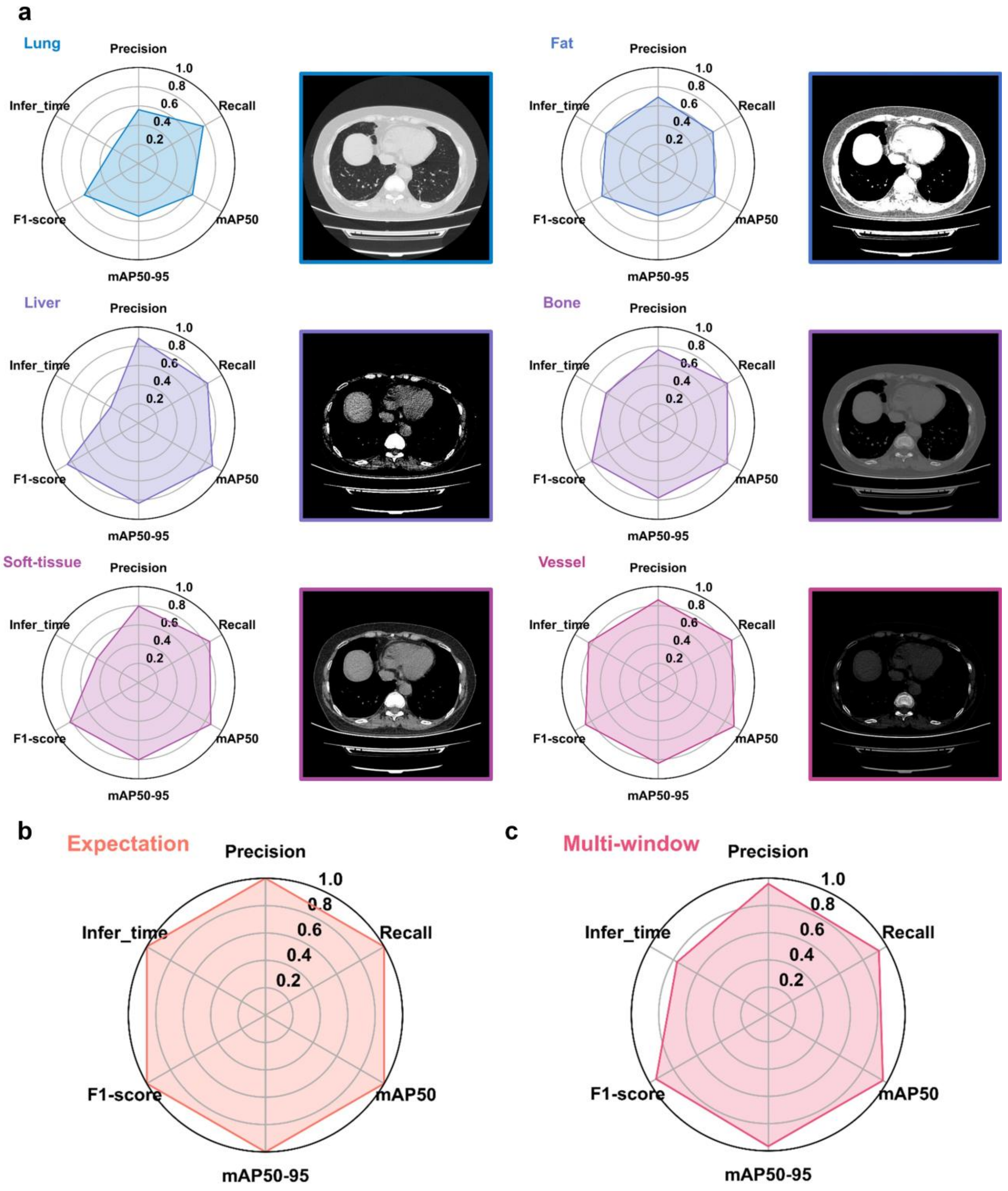

Fig. S2: Model performance improvement by multi-window enhancement methods. (a) Model metrics and their dataset performance under six window widths (WW) and window levels (WL), lung: 1500WW, -500WL; fat: 200WW, -100WL; liver: 120WW, 60WL; bone: 2000WW, 300WL; soft-tissue: 400WW, 40WL; and vessel: 700WW, 300WL. **(b)** Expected model performance. **(c)** Multi-window enhanced model performance.

Section 3 CSR and 3D images of ribs

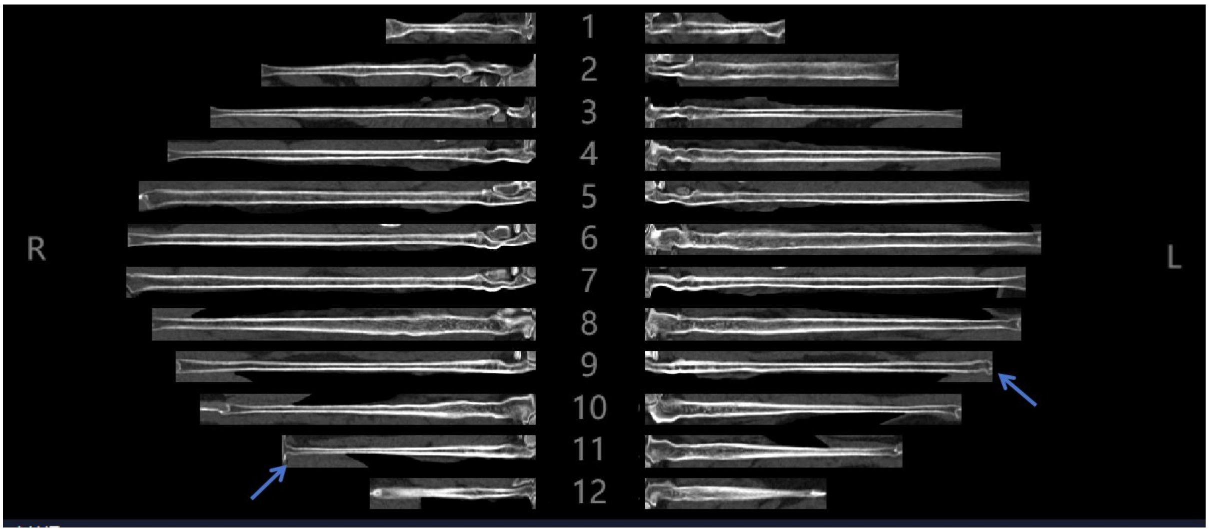

Fig. S3: CSR image of a patient with blue arrows showing reconstructed edge jaggedness.

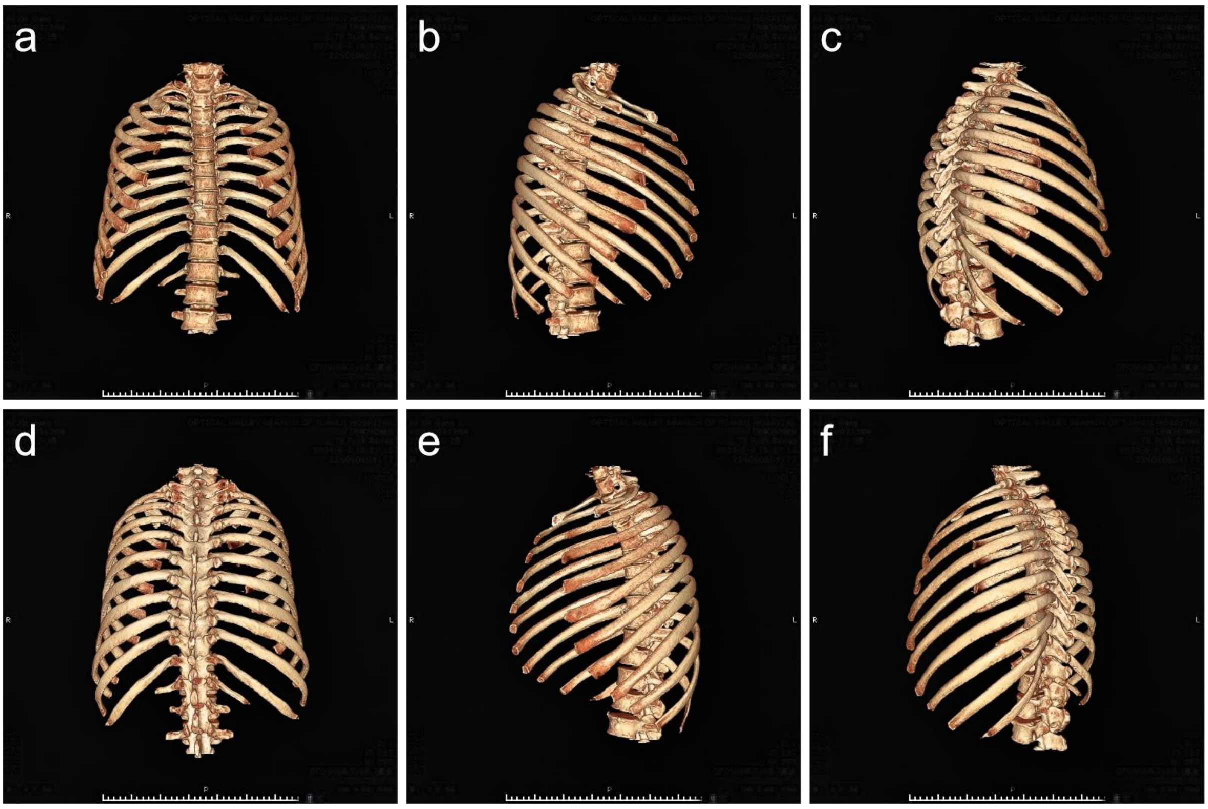

Fig. S4: 3D reconstructed images of the ribs of the same patient.

Section 4 MPR images with incomplete representation of ribs

Complete rib reconstruction is crucial for diagnosis, and incomplete reconstruction may lead to missed fractures, and during the diagnostic process in this study the reads were asked to mark whether the rib reconstruction was complete or not. An example of a complete reconstruction (Fig. S5) and an example of an incomplete reconstruction (Fig. S6) are shown below.

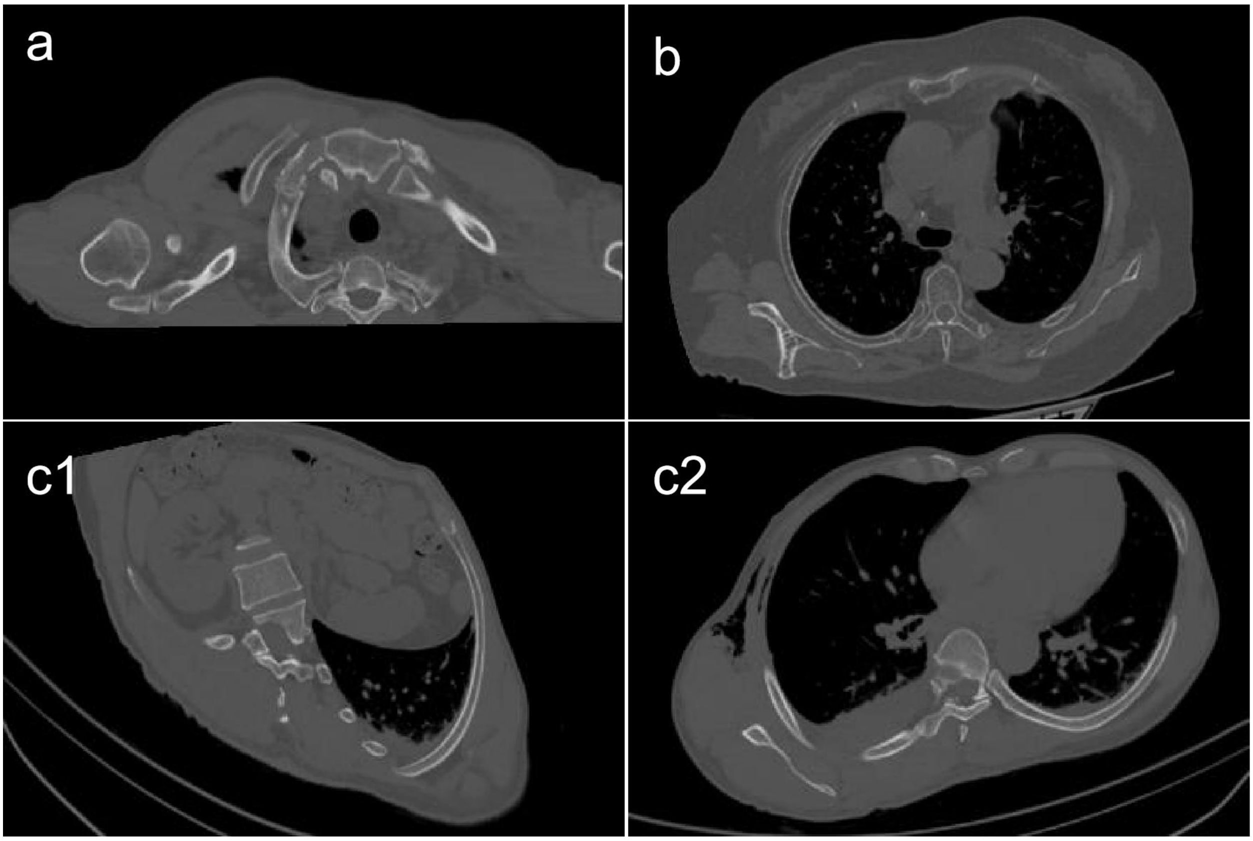

Fig. S5: (a) and **(b)** are single-figure reconstructions of the complete right 1st and 4th ribs, and **(c1)** and **(c2)** are two-figure reconstructions of the complete left 6th rib. As presented in the figure is considered by the reader to be reconstructed completely.

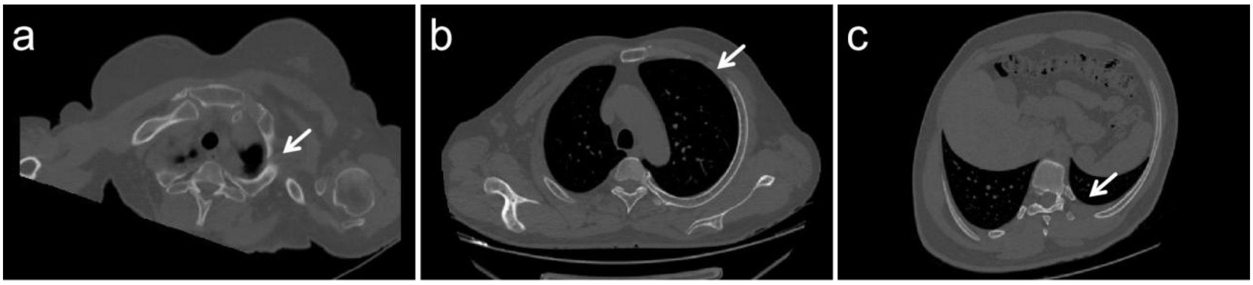

Fig. S6: Incomplete reconstruction of ribs (white arrows). (a) The loss of the middle of the first rib on the right side. **(b)** The partial absence of the anterior rib. **(c)** The partial absence of the posterior rib. All of the above is defined as incomplete reconstruction.

Section 5 MPR and cross-sectional images of false-positive cases

As shown in Fig. S7-12, 49 false-positive cases were identified (29 on the left side and 20 on the right). The main causes of false positives include: misinterpretation of ossified costal cartilage as rib fractures, uneven cortical bone density in localized areas of reconstruction images, and reconstruction artifacts caused by post-traumatic pleural effusion.

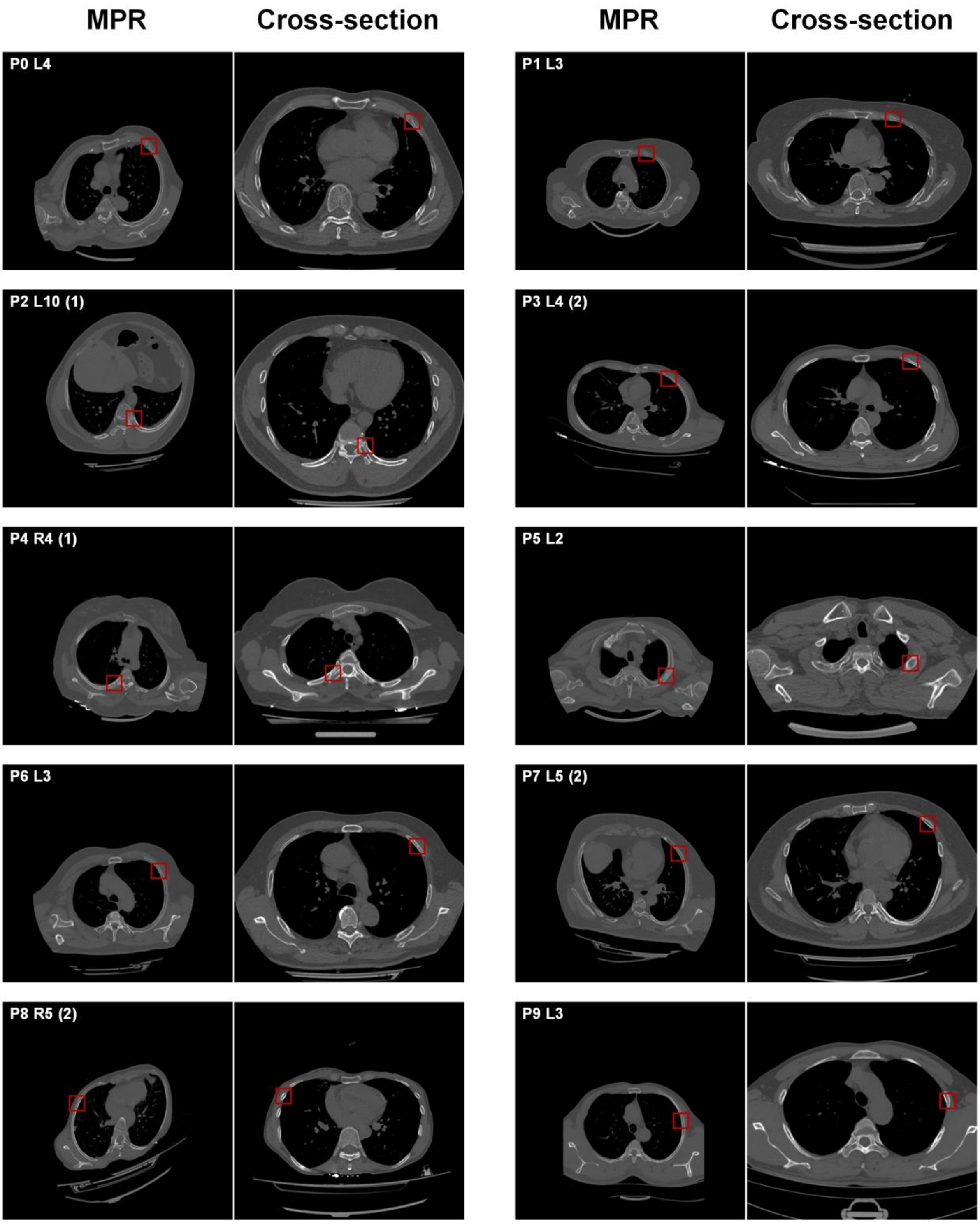

Fig. S7: MPR and cross-sectional images of 10 false-positive cases: Patient No.0-9.

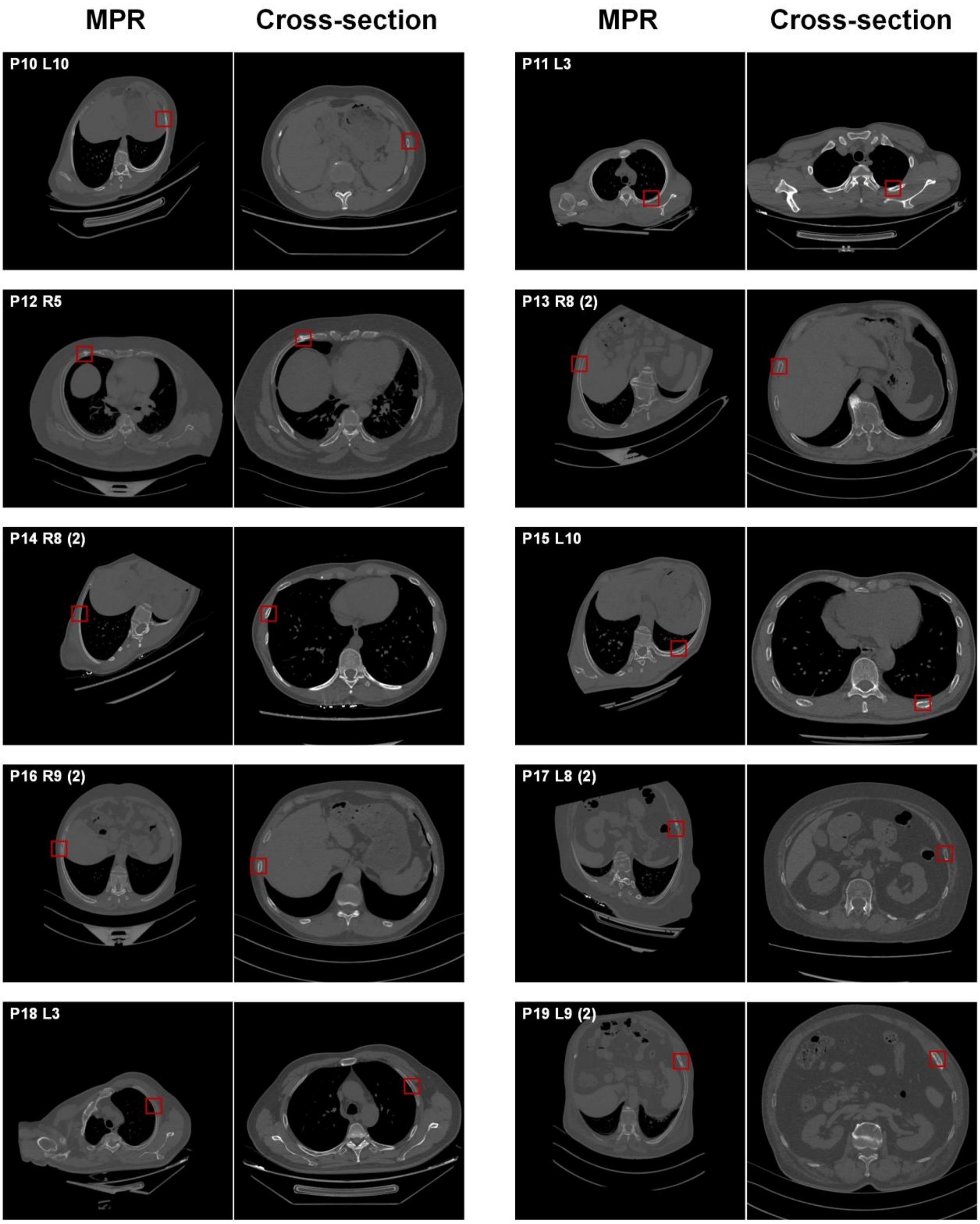

Fig. S8: MPR and cross-sectional images of 10 false-positive cases: Patient No.10-19.

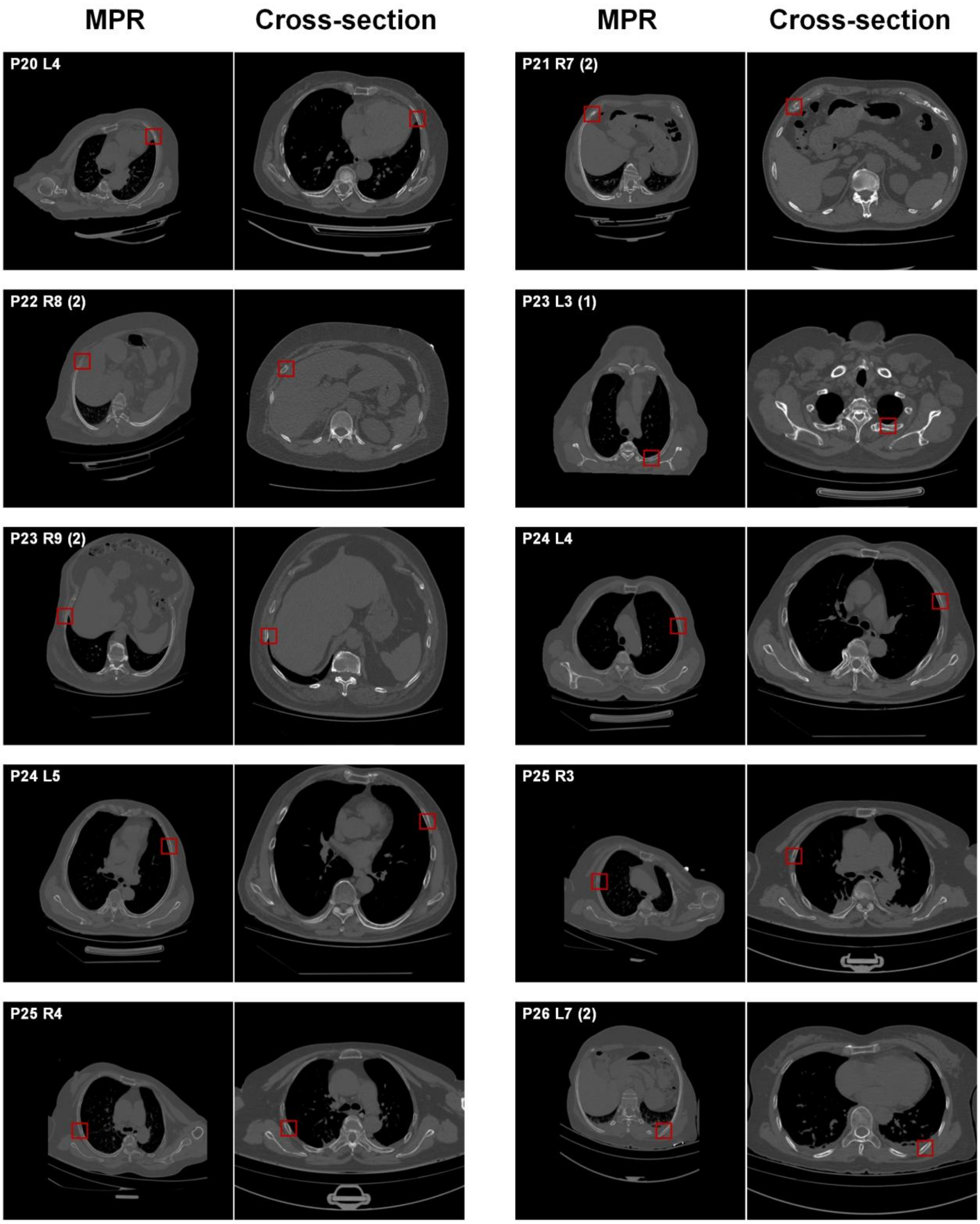

Fig. S9: MPR and cross-sectional images of 10 false-positive cases: Patient No.20-26.

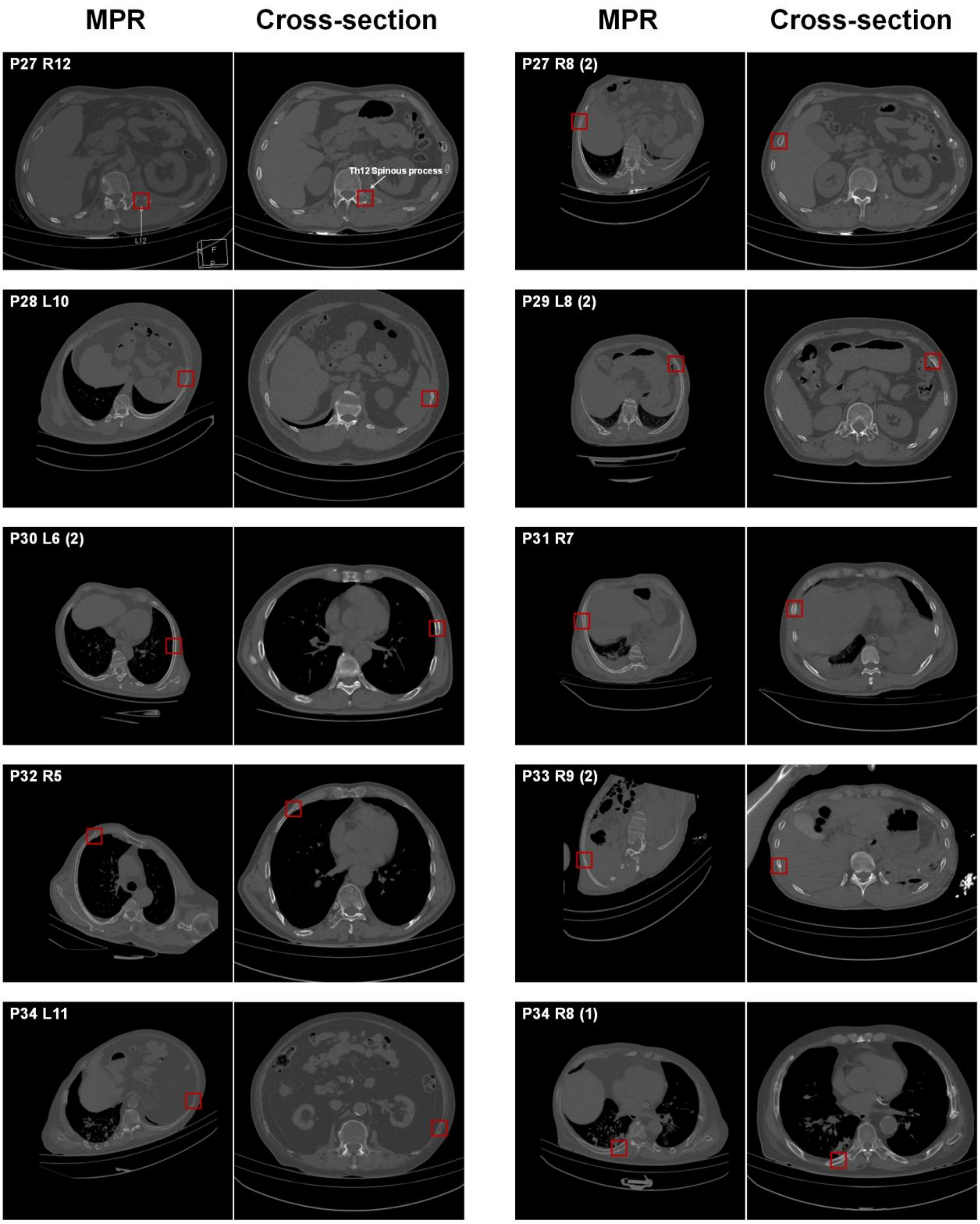

Fig. S10: MPR and cross-sectional images of 10 false-positive cases: Patient No.27-34.

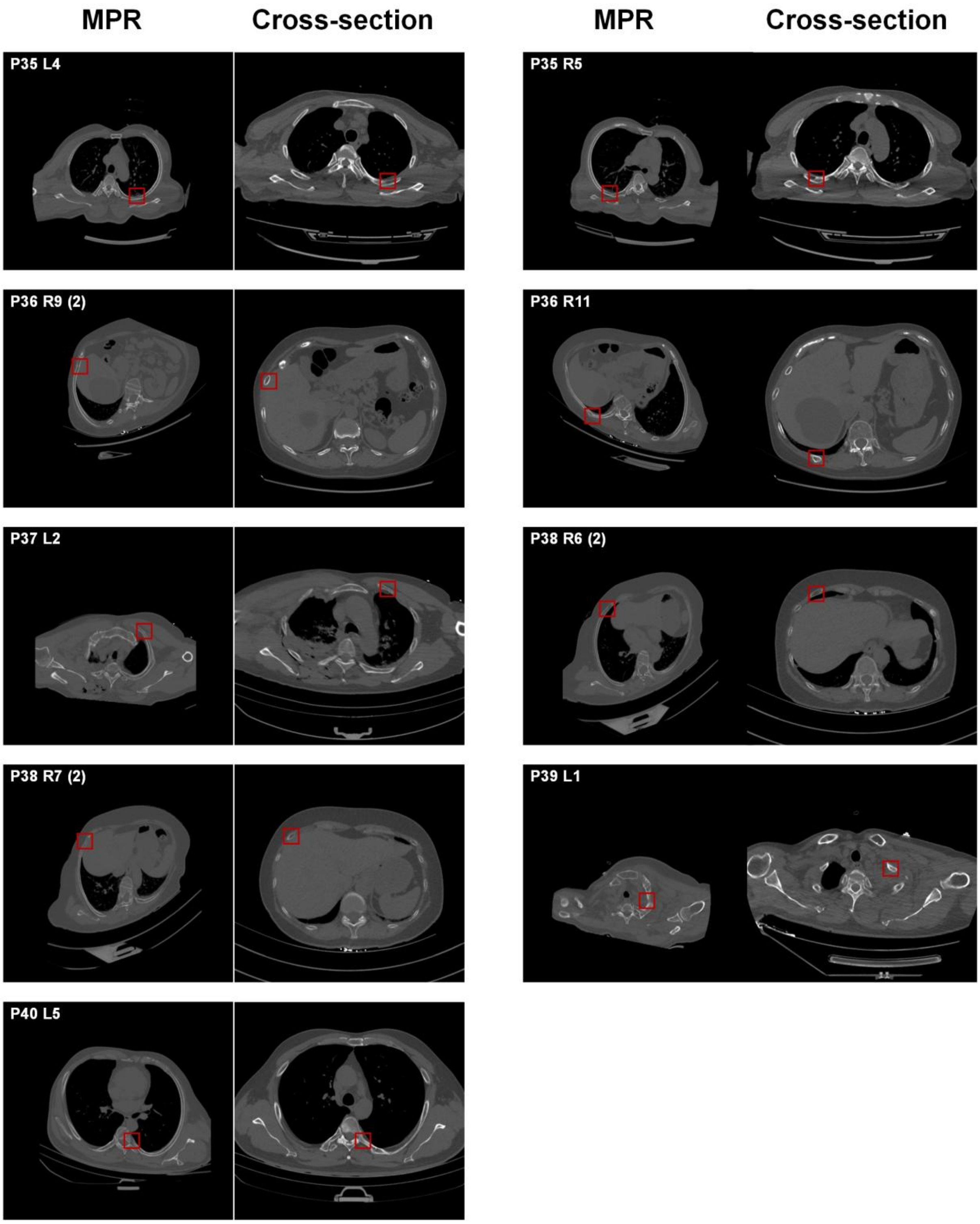

Fig. S11: MPR and cross-sectional images of 10 false-positive cases: Patient No.35-40.

Section 6 Sensitivity and specificity using different methods at per-rib level

Table S3: Sensitivity and specificity evaluated using different methods at the left, right and per-level of ribs level

		MPR image		Original CT image		CSR image		3D image	
		Se (95% CI)	Sp (95% CI)	Se (95% CI)	Sp (95% CI)	Se (95% CI)	Sp (95% CI)	Se (95% CI)	Sp (95% CI)
Left	1st	75.0% (40.9-92.9%)	99.5% (97.5-99.9%)	100.0% (67.6-100.0%)	99.5% (97.5-99.9%)	100.0% (67.6-100.0%)	99.5% (97.5-99.9%)	100.0% (67.6-100.0%)	99.5% (97.5-99.9%)
	2nd	58.6% (40.7-74.5%)	99.0% (96.4-99.7%)	86.2% (69.4-94.5%)	100.0% (98.1-100.0%)	86.2% (69.4-94.5%)	100.0% (98.1-100.0%)	86.2% (69.4-94.5%)	100.0% (98.1-100.0%)
	3rd	84.7% (73.5-91.8%)	96.5% (92.6-98.4%)	93.2% (83.8-97.3%)	100.0% (97.8-100.0%)	93.2% (83.8-97.3%)	100.0% (97.8-100.0%)	93.2% (83.8-97.3%)	100.0% (97.8-100.0%)
	4th	88.2% (78.5-93.9%)	96.9% (93.0-98.7%)	95.6% (87.8-98.5%)	98.1% (94.7-99.4%)	95.6% (87.8-98.5%)	98.1% (94.7-99.4%)	95.6% (87.8-98.5%)	98.1% (94.7-99.4%)
	5th	91.6% (83.6-95.9%)	98.0% (94.2-99.3%)	95.1% (88.3-98.1%)	98.6% (95.2-99.6%)	95.1% (88.3-98.1%)	98.6% (95.2-99.6%)	95.1% (88.3-98.1%)	98.6% (95.2-99.6%)
	6th	88.8% (79.6-94.3%)	99.4% (96.5-99.9%)	95.8% (88.5-98.6%)	100.0% (97.6-100.0%)	95.8% (88.5-98.6%)	100.0% (97.6-100.0%)	95.8% (88.5-98.6%)	100.0% (97.6-100.0%)
	7th	84.5% (74.3-91.1%)	100.0% (97.6-100.0%)	91.5% (82.8-96.1%)	100.0% (97.6-100.0%)	91.5% (82.8-96.1%)	100.0% (97.6-100.0%)	91.5% (82.8-96.1%)	100.0% (97.6-100.0%)
	8th	86.2% (75.1-92.8%)	98.3% (95.0-99.4%)	96.6% (88.3-99.0%)	99.4% (96.8-99.9%)	96.6% (88.3-99.0%)	99.4% (96.8-99.9%)	96.6% (88.3-99.0%)	99.4% (96.8-99.9%)
	9th	89.6% (77.8-95.5%)	98.9% (96.1-99.7%)	93.8% (83.2-97.9%)	100.0% (97.9-100.0%)	93.8% (83.2-97.9%)	100.0% (97.9-100.0%)	93.8% (83.2-97.9%)	100.0% (97.9-100.0%)
	10th	92.0% (75.0-97.8%)	98.0% (95.1-99.2%)	92.0% (75.0-97.8%)	99.5% (97.3-99.9%)	92.0% (75.0-97.8%)	99.5% (97.3-99.9%)	92.0% (75.0-97.8%)	99.5% (97.3-99.9%)
	11th	88.2% (65.7-96.7%)	100.0% (98.2-100.0%)	94.1% (73.0-99.0%)	100.0% (98.2-100.0%)	94.1% (73.0-99.0%)	100.0% (98.2-100.0%)	94.1% (73.0-99.0%)	100.0% (98.2-100.0%)
	12th	100.0% (70.1-100.0%)	99.1% (96.8-99.8%)	100.0% (70.1-100.0%)	99.5% (97.5-99.9%)	100.0% (70.1-100.0%)	99.5% (97.5-99.9%)	100.0% (70.1-100.0%)	99.5% (97.5-99.9%)

Right	1st	70.0% (39.7-89.2%)	100.0% (98.3-100.0%)	80.0% (49.0-94.3%)	100.0% (98.3-100.0%)	80% (49.0-94.3%)	100.0% (98.3-100.0%)	80.0% (49.0-94.3%)	100.0% (98.3-100.0%)
	2nd	58.3% (38.8-75.5%)	100.0% (98.2-100.0%)	87.5% (69.0-95.7%)	99.5% (97.3-99.9%)	87.5% (69-95.7%)	99.5% (97.3-99.9%)	87.5% (69.0-95.7%)	99.5% (97.3-99.9%)
	3rd	86.2% (75.1-92.8%)	99.4% (96.8-99.9%)	96.6% (88.3-99.0%)	99.4% (96.8-99.9%)	96.6% (88.3-99%)	99.4% (96.8-99.9%)	96.6% (88.3-99.0%)	99.4% (96.8-99.9%)
	4th	81.9% (71.5-89.1%)	98.7% (95.5-99.7%)	95.8% (88.5-98.6%)	99.3% (96.5-99.9%)	95.8% (88.5-98.6%)	99.3% (96.5-99.9%)	95.8% (88.5-98.6%)	99.3% (96.5-99.9%)
	5th	85.4% (76.1-91.4%)	97.3% (93.3-98.9%)	96.3% (89.8-98.7%)	98.6% (95.2-99.6%)	96.3% (89.8-98.7%)	98.6% (95.2-99.6%)	96.3% (89.8-98.7%)	98.6% (95.2-99.6%)
	6th	91.4% (82.5-96.0%)	99.4% (96.5-99.9%)	97.1% (90.2-99.2%)	100.0% (97.7-100.0%)	97.1% (90.2-99.2%)	100.0% (97.7-100.0%)	97.1% (90.2-99.2%)	100.0% (97.7-100.0%)
	7th	85.7% (75.0-92.3%)	98.2% (94.9-99.4%)	95.2% (86.9-98.4%)	100.0% (97.8-100.0%)	95.2% (86.9-98.4%)	100.0% (97.8-100.0%)	95.2% (86.9-98.4%)	100.0% (97.8-100.0%)
	8th	86.7% (73.8-93.7%)	97.8% (94.6-99.2%)	93.3% (82.1-97.7%)	99.5% (97.0-99.9%)	93.3% (82.1-97.7%)	99.5% (97.0-99.9%)	93.3% (82.1-97.7%)	99.5% (97.0-99.9%)
	9th	87.8% (74.5-94.7%)	97.9% (94.7-99.2%)	90.2% (77.5-96.1%)	99.5% (97.1-99.9%)	90.2% (77.5-96.1%)	99.5% (97.1-99.9%)	90.2% (77.5-96.1%)	99.5% (97.1-99.9%)
	10th	77.8% (59.2-89.4%)	100.0% (98.1-100.0%)	88.9% (71.9-96.1%)	99.5% (97.3-99.9%)	88.9% (71.9-96.1%)	99.5% (97.3-99.9%)	88.9% (71.9-96.1%)	99.5% (97.3-99.9%)
	11th	92.9% (68.5-98.7%)	99.5% (97.4-99.9%)	92.9% (68.5-98.7%)	100.0% (98.3-100.0%)	92.9% (68.5-98.7%)	100.0% (98.3-100.0%)	92.9% (68.5-98.7%)	100.0% (98.3-100.0%)
	12th	83.3% (55.2-95.3%)	100.0% (98.3-100.0%)	91.7% (64.6-98.5%)	100.0% (98.3-100.0%)	91.7% (64.6-98.5%)	100.0% (98.3-100.0%)	91.7% (64.6-98.5%)	100.0% (98.3-100.0%)

Section 7 Error distribution in the number of rib fractures by different methods

Table S4: Error distribution in the number of rib fractures by different methods at the per-patient level.

Error	MPR image	Original CT image	CSR image	3D image
2*	10.0%	0.9%	8.3%	15.2%
1	26.1%	20.0%	20.4%	25.7%
0	49.6%	75.2%	33.9%	26.1%
-1	9.1%	3.5%	18.7%	3.0%
-2	1.7%	0.0%	8.7%	0.4%

*Compared with the reference standard, error values of 1 and 2 indicate underestimation (missed rib fractures), 0 denotes exact agreement, and error values of −1 and −2 indicate overestimation (false-positive rib fractures).

Section 8 Statistical analysis of significance testing

Table S5: Pairwise McNemar p-values across rib levels and reconstruction methods.

	MPR vs Original	MPR vs CSR	MPR vs 3D	CSR vs Original	CSR vs 3D	3D vs Original
Rib 1	**0.25 ***	**1.000***	1.95×10^{-3}	**0.125***	3.91×10^{-3}	2.44×10^{-4}
Rib 2	3.05×10^{-5}	**0.125***	**0.25***	1.91×10^{-6}	**1.000***	3.81×10^{-6}
Rib 3	1.53×10^{-5}	9.31×10^{-10}	5.96×10^{-8}	7.11×10^{-15}	3.12×10^{-2}	4.55×10^{-13}
Rib 4	7.63×10^{-6}	7.45×10^{-9}	1.16×10^{-10}	2.84×10^{-14}	3.12×10^{-2}	4.44×10^{-16}
Rib 5	6.10×10^{-5}	9.54×10^{-7}	1.16×10^{-10}	2.91×10^{-11}	2.44×10^{-4}	3.55×10^{-15}
Rib 6	9.77×10^{-4}	2.98×10^{-8}	3.55×10^{-15}	1.46×10^{-11}	2.38×10^{-7}	1.73×10^{-18}
Rib 7	1.22×10^{-4}	9.31×10^{-10}	9.09×10^{-13}	5.68×10^{-14}	1.95×10^{-3}	5.55×10^{-17}
Rib 8	1.22×10^{-4}	9.77×10^{-4}	2.98×10^{-8}	5.96×10^{-8}	6.10×10^{-5}	1.82×10^{-12}
Rib 9	7.81×10^{-3}	7.63×10^{-6}	4.66×10^{-10}	2.98×10^{-8}	1.22×10^{-4}	1.82×10^{-12}
Rib 10	**0.0625***	9.77×10^{-4}	4.88×10^{-4}	3.05×10^{-5}	**1.000***	1.53×10^{-5}
Rib 11	**0.500***	**0.125***	1.56×10^{-2}	3.12×10^{-2}	**0.25***	3.91×10^{-3}

Rib 12	**0.500***	**1.000***	**0.500***	**0.500***	**0.500***	**0.125***
Overall	9.40×10^{-38}	4.08×10^{-56}	6.41×10^{-67}	9.59×10^{-94}	6.94×10^{-6}	1.55×10^{-120}

* notation for non-significant differences.

Table S6: Pairwise McNemar p-values across fracture types and reconstruction methods.

	MPR vs Original	MPR vs CSR	MPR vs 3D	CSR vs Original	CSR vs 3D	3D vs Original
Displaced fractures	**0.146***	7.18×10^{-14}	2.37×10^{-21}	2.05×10^{-15}	6.65×10^{-4}	6.90×10^{-23}
Non-displaced fractures	7.63×10^{-5}	3.23×10^{-27}	1.50×10^{-17}	2.47×10^{-32}	**0.864***	2.04×10^{-21}
Buckle fractures	1.52×10^{-12}	2.92×10^{-41}	1.19×10^{-12}	1.32×10^{-52}	7.39×10^{-8}	2.22×10^{-25}
Old fractures	1.49×10^{-8}	2.19×10^{-66}	1.20×10^{-13}	1.16×10^{-67}	2.30×10^{-30}	1.02×10^{-15}

* notation for non-significant differences.

Table S7: Statistical comparison of diagnostic sensitivity across sex and age groups for each reconstruction method.

	MPR image	Original CT image	CSR image	3D image
Sex (Fisher's exact test)	**0.588***	**0.528***	**0.220***	**0.276***
Age (Cochran-Armitage test)	**0.086***	4.29×10^{-4}	1.50×10^{-4}	1.38×10^{-5}

* notation for non-significant differences.
